# Supplementary material for: Nedd4 ubiquitylates VDAC2/3 to suppress erastin-induced ferroptosis in melanoma
Source: Nat Commun. 2020 Jan 23;11:433. doi: 10.1038/s41467-020-14324-x (PMC6978386; doi:10.1038/s41467-020-14324-x)
Supplement: Supplementary file 1 — Supplementary Information [file 41467_2020_14324_MOESM1_ESM.pdf]

Supplementary Information

Nedd4 ubiquitylates VDAC2/3 to suppress erastin-induced ferroptosis in melanoma.

Yang, et al.

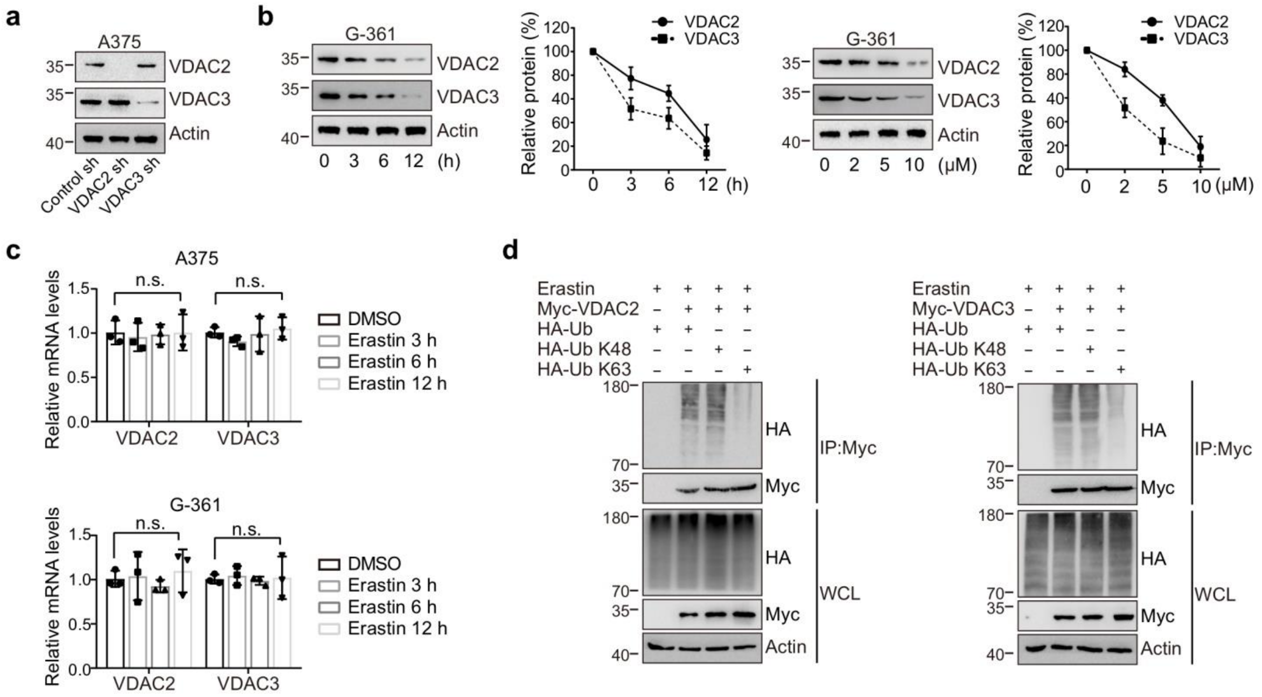

Supplementary Figure 1. Erastin treatment promotes the ubiquitin-proteasomal degradation of VDAC2/3 in melanoma cells.

(**a**) VDAC-isoform-specific shRNAs are selective in knockdown of protein. (**b**) The protein level of VDAC2/3 was downregulated in response to erastin in melanoma cells. G-361 cells were treated with erastin at the indicated time (0-12 h, 10 μM) or indicated concentrations (0-10 μM, 12 h). The protein level of VDAC2/3 was analyzed by immunoblotting and quantitated by Image J. (**c**) The mRNA level of VDAC2/3 was not changed in response to erastin in melanoma cells. A375 and G-361 cells were treated with erastin (5 μM for A375, 10 μM for G-361). The mRNA level of VDAC2/3 was quantified by qRT-PCR. (**d**) Erastin promotes the K48 ubiquitination of VDAC2/3. A375 cells were transfected with indicated DNA constructs. After 48 h transfection, cells were treated with DMSO or erastin (5 μM) for 8 h, then MG132 (50 mM) was added into the culture medium for an additional 4 h. Lysates from A375 cells were immunoprecipitated with anti-Myc. Immunoblot assays were performed to analyze the presence of indicated proteins and levels of ubiquitination. Actin was used as a loading control. Data shown represent mean ± SD from three independent experiments. Comparisons were made using Student’s t-test. n.s., not significant.

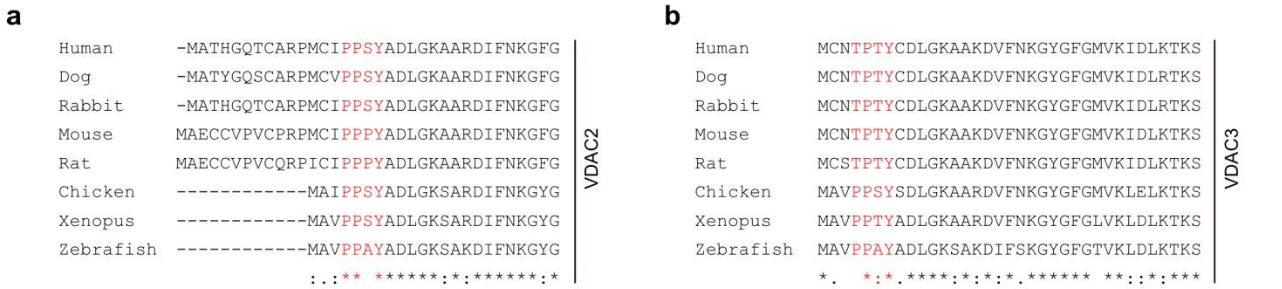

c

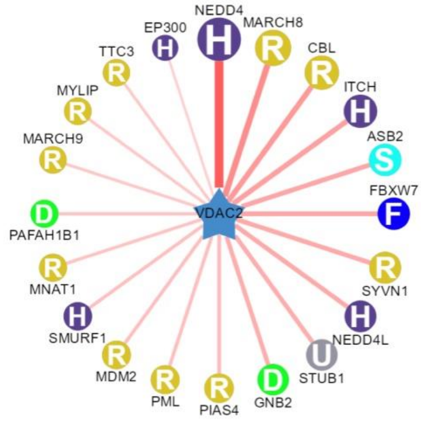

Rank	Gene symbol	Gene description	Score	Confidence level
1	NEDD4	E3 ubiquitin-protein ligase NEDD4	0.829	HIGH
2	MARCH8	E3 ubiquitin-protein ligase MARCH8	0.738	MIDDLE
3	CBL	E3 ubiquitin-protein ligase CBL	0.716	MIDDLE
4	ITCH	E3 ubiquitin-protein ligase Itchy homolog	0.705	MIDDLE
5	ASB2	Ankyrin repeat and SOCS box protein 2	0.694	MIDDLE
6	FBXW7	F-box/WD repeat-containing protein 7	0.694	MIDDLE
7	SYVN1	E3 ubiquitin-protein ligase synoviolin	0.687	MIDDLE
8	NEDD4L	E3 ubiquitin-protein ligase NEDD4-like	0.686	MIDDLE
9	STUB1	E3 ubiquitin-protein ligase CHIP	0.681	MIDDLE
10	GNB2	Guanine nucleotide-binding protein G(I)/G(S)/G(T) subunit beta-2	0.681	MIDDLE

d

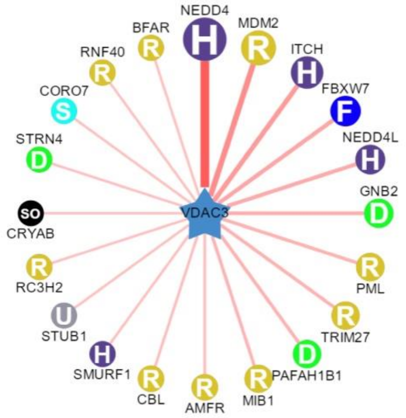

Rank	Gene symbol	Gene description	Score	Confidence level
1	NEDD4	E3 ubiquitin-protein ligase NEDD4	0.829	HIGH
2	MDM2	E3 ubiquitin-protein ligase Mdm2	0.738	MIDDLE
3	ITCH	E3 ubiquitin-protein ligase Itchy homolog	0.714	MIDDLE
4	FBXW7	F-box/WD repeat-containing protein 7	0.694	MIDDLE
5	NEDD4L	E3 ubiquitin-protein ligase NEDD4-like	0.686	MIDDLE
6	GNB2	Guanine nucleotide-binding protein G(I)/G(S)/G(T) subunit beta-2	0.681	MIDDLE
7	PML	Protein PML	0.671	MIDDLE
8	TRIM27	Zinc finger protein RFP	0.671	MIDDLE
9	PAFAH1B1	Platelet-activating factor acetylhydrolase IB subunit alpha	0.671	MIDDLE
10	MIB1	E3 ubiquitin-protein ligase MIB1	0.667	MIDDLE

Supplementary Figure 2. Identification of Nedd4 as the E3 ligase of VDAC2/3 by bioinformatics

(**a, b**) Sequence alignment of PPxY motifs of VDAC2 (a) and VDAC3 (b). The PPxY sequence of VDAC2 (red) is highly conserved in different species. The sequence of VDAC3 evolved from PPxY to TPxY. (**c, d**) Nedd4 is predicted as the specific E3 ligase of VDAC2 (c) and VDAC3 (d) by UbiBrowser database[1].

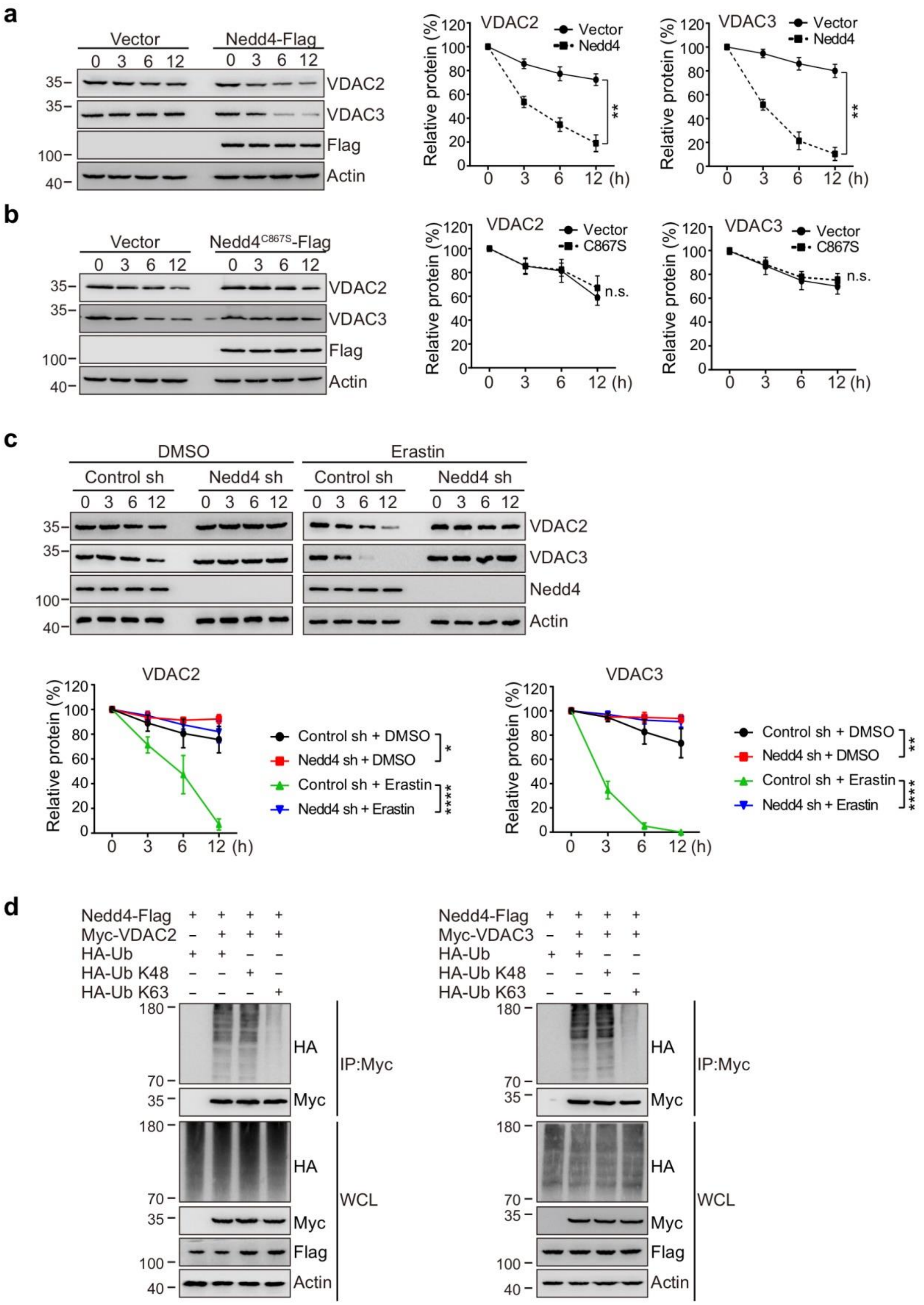

Supplementary Figure 3. Nedd4 promotes the degradation of VDAC2/3

(**a, b**) The wild type Nedd4 (a) decreases VDAC2/3 protein level, but the C867S mutant (b) cannot downregulate VDAC2/3. A375 cells expressing Flag-Nedd4 or Flag-Nedd4^{C867S} were treated with cycloheximide (CHX, 200 μg/ml) and erastin (0-12 h, 5 μM). The protein level of VDAC2/3 was analyzed by immunoblotting and quantitated by Image J. (**c**) Nedd4 is critical to the stability of

VDAC2/3 before or after erastin treatment. A375 cells expressing indicated shRNA constructs were treated with cycloheximide (CHX, 200 µg/ml) and erastin (0-12 h, 5 µM). The protein level of VDAC2/3 was analyzed by Immunoblotting and quantitated by Image J. (**d**) Nedd4 promotes the K48 ubiquitination of VDAC2/3. A375 cells were transfected with indicated DNA constructs. After 48 h transfection, cells were treated with DMSO or erastin (5 µM) for 8 h, then MG132 (50 mM) was added into the culture medium for an additional 4 h. Lysates from A375 cells were immunoprecipitated with anti-Myc. Immunoblot assays were performed to analyze the presence of indicated proteins and levels of ubiquitination. Actin was used as a loading control. Data shown represent mean ± SD from three independent experiments. Comparisons were made using Student's t-test. *, $p < 0.05$; **, $p < 0.01$; ****, $p < 0.0001$. n.s., not significant.

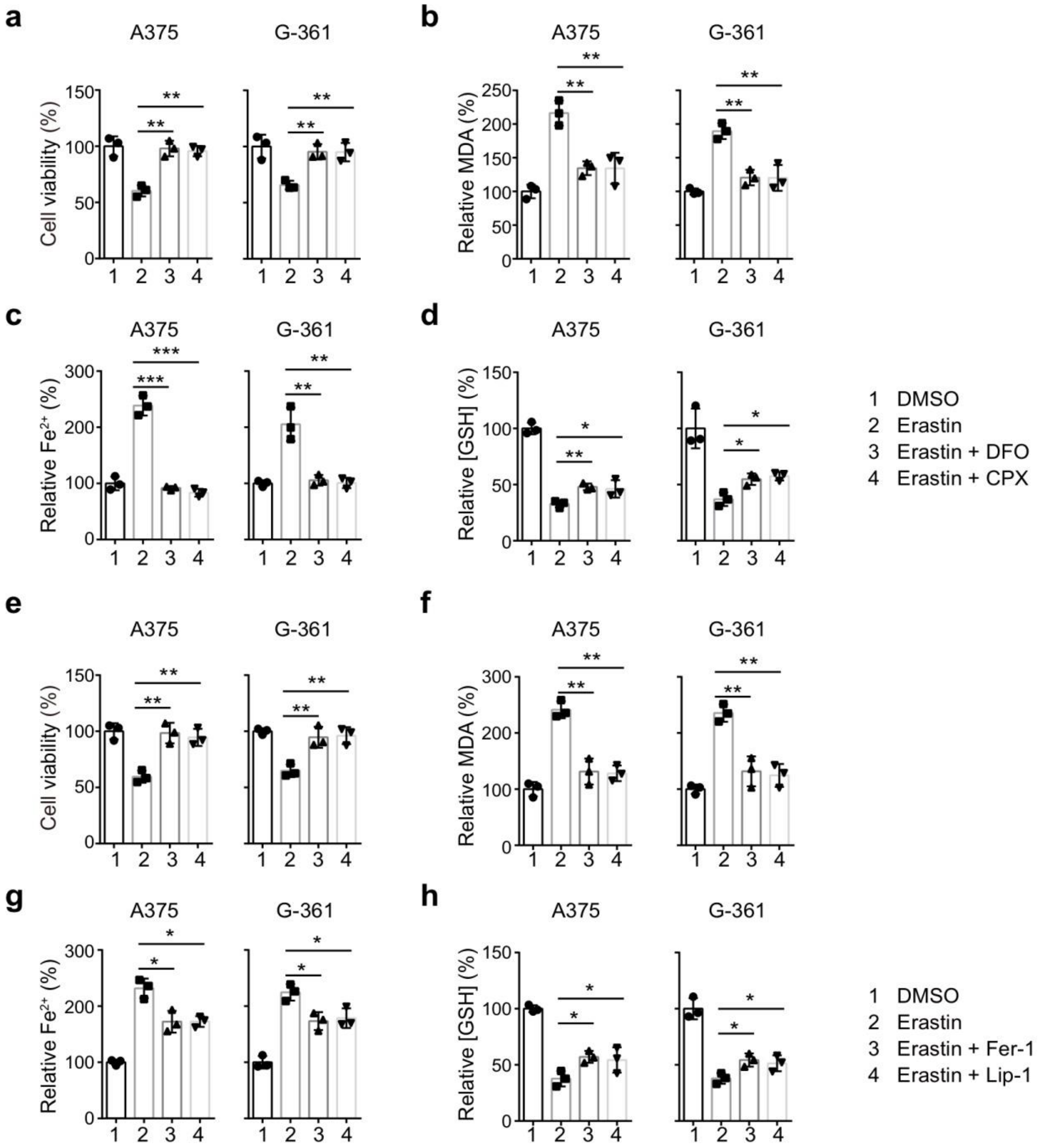

Supplementary Figure 4. Scavengers for Fe^{2+} and lipid ROS ablate erastin-induced ferroptosis in the presence of Nedd4 overexpression.

(**a-d**) A375 and G-361 cells were treated with erastin (5 μM for A375, 10 μM for G-361), erastin + DFO (100 μM), erastin + CPX (5 μM) for 12 h, and cell viability was assayed using a CCK8 kit (a), the lipid formation was measured by MDA assay (b), the accumulation of Fe^{2+} was measured by iron detection assay (c), the concentration of GSH was detected by relative assay kits (d). (**e-h**) A375 and G-361 cells were treated with erastin (5 μM for A375, 10 μM for G-361), erastin + Fer-1 (1 μM), erastin + Lip-1 (100 nM) for 12 h, and cell viability was assayed using a CCK8 kit (e), the lipid formation was measured by MDA assay (f), the accumulation of Fe^{2+} was measured by iron detection assay (g), the concentration of GSH was detected by relative assay kits (h). Data shown represent mean ± SD from three independent experiments. Comparisons were made using Student's t-test. *, $p < 0.05$; **, $p < 0.01$; ***, $p < 0.001$.

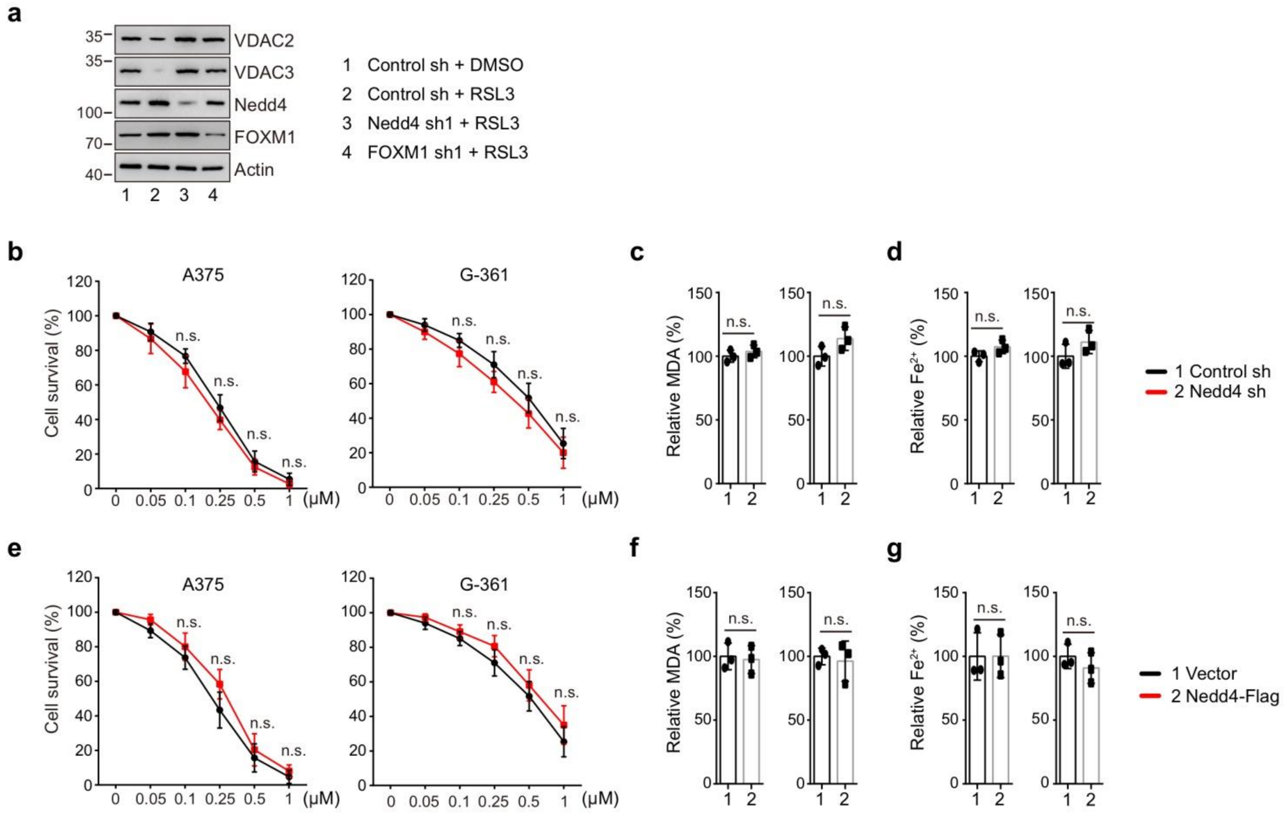

Supplementary Figure 5. Nedd4 has no significant effects on RSL3-induced ferroptosis in melanoma cells.

(**a**) Knockdown of FOXM1 or Nedd4 suppressed RSL3-induced VDAC2/3 degradation. A375 cells expressing indicated shRNA constructs were treated with DMSO or RSL3 (0.25 μM) for 24 h, the protein levels of VDAC2, VDAC3, Nedd4, and FOXM1 were assayed by western blot. (**b-d**) Knockdown of Nedd4 cannot enhance RSL3-induced ferroptotic cell death. Indicated cells were treated with RSL3 (0.05-1 μM) for 24 h, and cell viability was assayed using a CCK8 kit (b). Indicated cells were treated with RSL3 (0.25 μM) for 24 h, the lipid formation was measured by MDA assay (c), and the accumulation of Fe^{2+} was measured by iron detection assay (d). (**e-g**) Overexpression of Nedd4 cannot suppress RSL3-induced ferroptotic cell death. Indicated cells were treated with RSL3 (0.05-1 μM) for 24 h, and cell viability was assayed using a CCK8 kit (e). Indicated cells were treated with RSL3 (0.25 μM) for 24 h, the lipid formation was measured by MDA assay (f), and the accumulation of Fe^{2+} was measured by iron detection assay (g). Data shown represent mean ± SD from three independent experiments. Comparisons were made using Student's t-test. n.s., not significant.

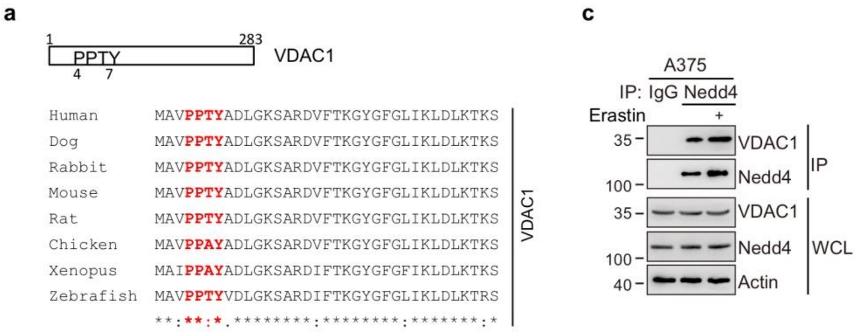

b

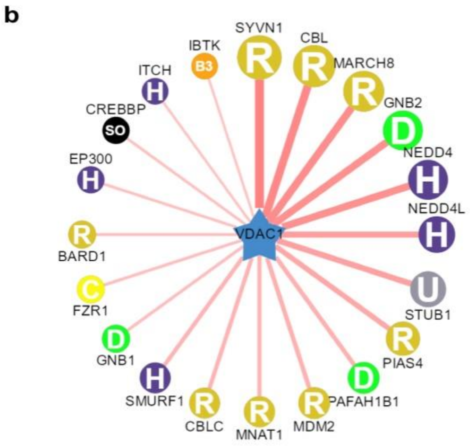

Rank	Gene symbol	Gene description	Score	Confidence level
1	SYVN1	E3 ubiquitin-protein ligase synoviolin	0.762	HIGH
2	CBL	E3 ubiquitin-protein ligase CBL	0.747	MIDDLE
3	MARCH8	E3 ubiquitin-protein ligase MARCH8	0.738	MIDDLE
4	GNB2	Guanine nucleotide-binding protein G(I)/G(S)/G(T) subunit beta-2	0.732	MIDDLE
5	NEDD4	E3 ubiquitin-protein ligase NEDD4	0.732	MIDDLE
6	NEDD4L	E3 ubiquitin-protein ligase NEDD4-like	0.710	MIDDLE
7	STUB1	E3 ubiquitin-protein ligase CHIP	0.704	MIDDLE
8	PIAS4	E3 SUMO-protein ligase PIAS4	0.692	MIDDLE
9	PAFAH1B1	Platelet-activating factor acetylhydrolase IB subunit alpha	0.676	MIDDLE
10	MDM2	E3 ubiquitin-protein ligase Mdm2	0.676	MIDDLE

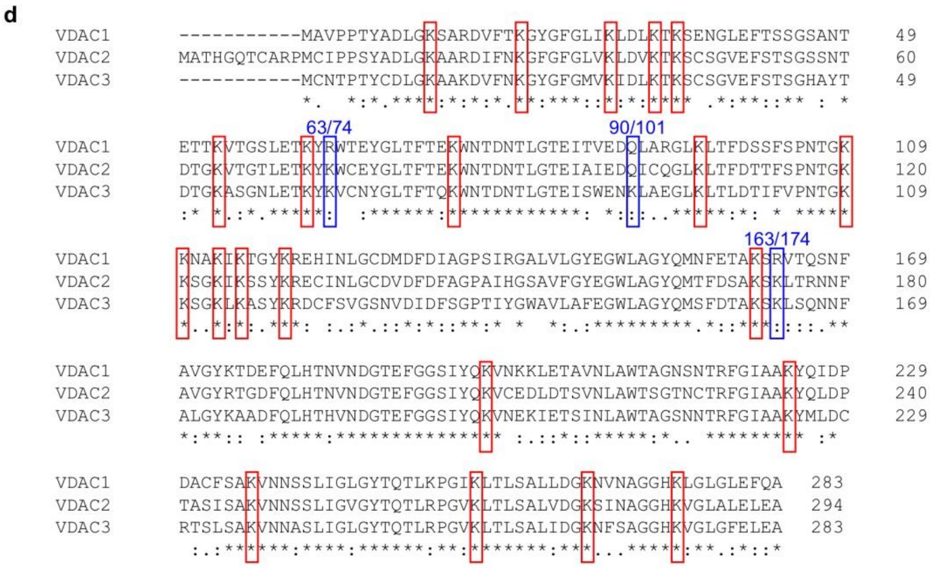

Supplementary Figure 6. Nedd4 interacts with VDAC1 in melanoma cells

(**a**) Sequence alignment of PPxY motifs of VDAC1. The PPxY sequence of VDAC1 (red) is highly conserved in different species. (**b**) Nedd4 is predicted as an E3 ligase of VDAC1 by UbiBrowser database[1]. (**c**) Interaction between endogenous VDAC1 and Nedd4 under basal conditions and erastin treatment. A375 cells were treated with DMSO or erastin (5 µM) for 8 h, then MG132 (50 mM) was added into the culture medium for 4 h. Whole cell lysates (WCL) were used for IP with control serum (IgG) or anti-Nedd4 antibody, followed by immunoblotting (IB) with the indicated antibodies.

The bottom panel shows endogenous protein expression with actin as a loading control. (**d**) Alignment of the full length protein sequences of VDAC1, VDAC2, and VDAC3. The conserved K sites were marked with red color, and the K to R/Q mutations in VDAC1 were marked with blue color (R63, Q90, and R163 in VDAC1; K74, Q101, and K174 in VDAC2; K63, K90, and K163 in VDAC3).

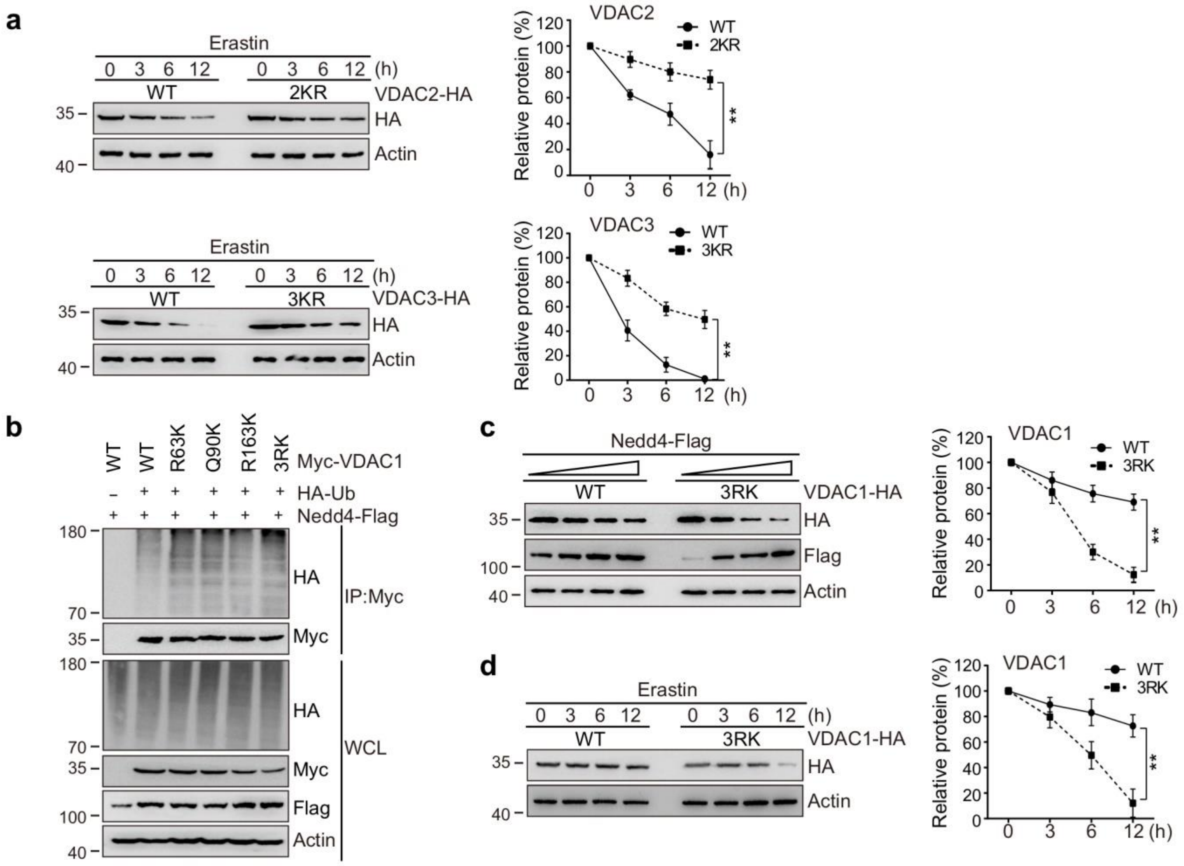

Supplementary Figure 7. The three non-conserved K sites mediate the stabilities of VDAC isoforms

(**a**) A375 cells were transfected with combinations of DNA constructs as indicated and treated with erastin (5 μM). Cell lysates were analyzed by immunoblotting with indicated antibodies. The protein level of VDAC2/3 was quantitated by Image J and normalized to actin. (**b**) A375 cells were transfected with combinations of DNA constructs as indicated and treated with MG132 for 4 h. Lysates were immunoprecipitated with anti-Myc, and western blots were performed to analyze the presence of indicated proteins and levels of ubiquitination. (**c**) A375 cells were transfected with combinations of DNA constructs as indicated. Cell lysates were analyzed by immunoblotting with indicated antibodies. The protein level of VDAC1 was quantitated by Image J and normalized to actin levels. (**d**) A375 cells were transfected with indicated DNA constructs and treated with erastin (5 μM). Cell lysates were analyzed by immunoblotting with indicated antibodies. The protein level of VDAC1 was quantitated by Image J and normalized to actin levels. Data shown represent mean ± SD from three independent experiments. Comparisons were made using one-way ANOVA.**, $p < 0.01$.

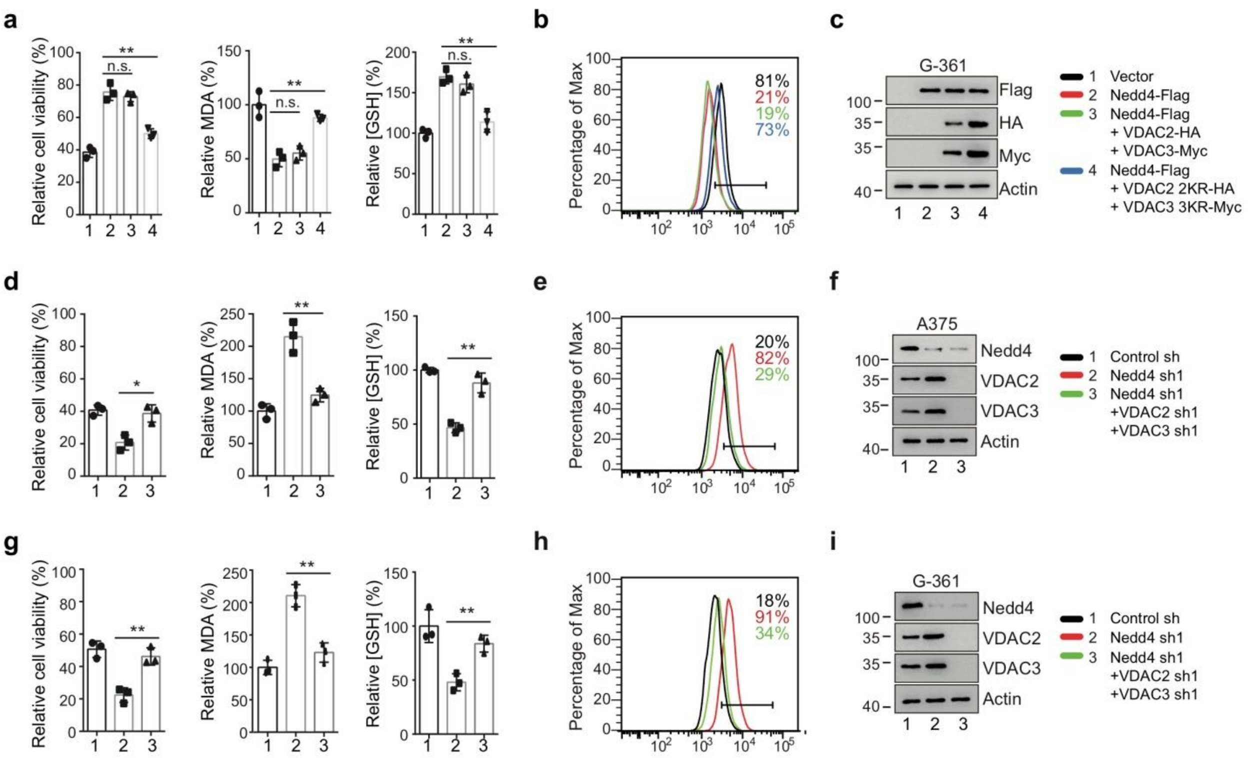

Supplementary Figure 8. VDAC2/3 partially rescued the cell sensitivity to erastin which was regulated by Nedd4.

(**a-c**) Overexpression of KR mutants of VDAC2/3 increased erastin-induced ferroptosis which was suppressed by Nedd4. G-361 cells were transfected with indicated DNA constructs for 48 h, and treated with erastin (10 µM) for 12 h. Cell viability, intracellular MDA, and GSH levels were measured (a). The lipid ROS level was assessed by flow cytometry using C11-BODIPY (b). The protein levels of overexpressed constructs were assessed by immunoblotting (c). (**d-f**) Depletion of VDAC2/3 suppressed erastin-induced ferroptosis which was enhanced by Nedd4 knockdown. A375 cells were transfected with indicated DNA constructs for 48 h, and treated with erastin (5 µM) for 12 h. Cell viability, intracellular MDA, GSH, and GSSG levels were measured (d). The lipid ROS level was assessed by flow cytometry using C11-BODIPY (e). The protein levels of indicated genes were assessed by immunoblotting (f). (**g-i**) Depletion of VDAC2/3 suppressed erastin-induced ferroptosis which was enhanced by Nedd4 knockdown. G-361 cells were transfected with indicated DNA constructs for 48 h, and treated with erastin (10 µM) for 12 h. Cell viability, intracellular MDA, GSH, and GSSG levels were measured (g). The lipid ROS level was assessed by flow cytometry using C11-BODIPY (h). The protein levels of indicated genes were assessed by immunoblotting (i). Data shown represent mean ± SD from three independent experiments. Comparisons were made using Student's t-test. *, $p < 0.05$; **, $p < 0.01$; n.s., not significant.

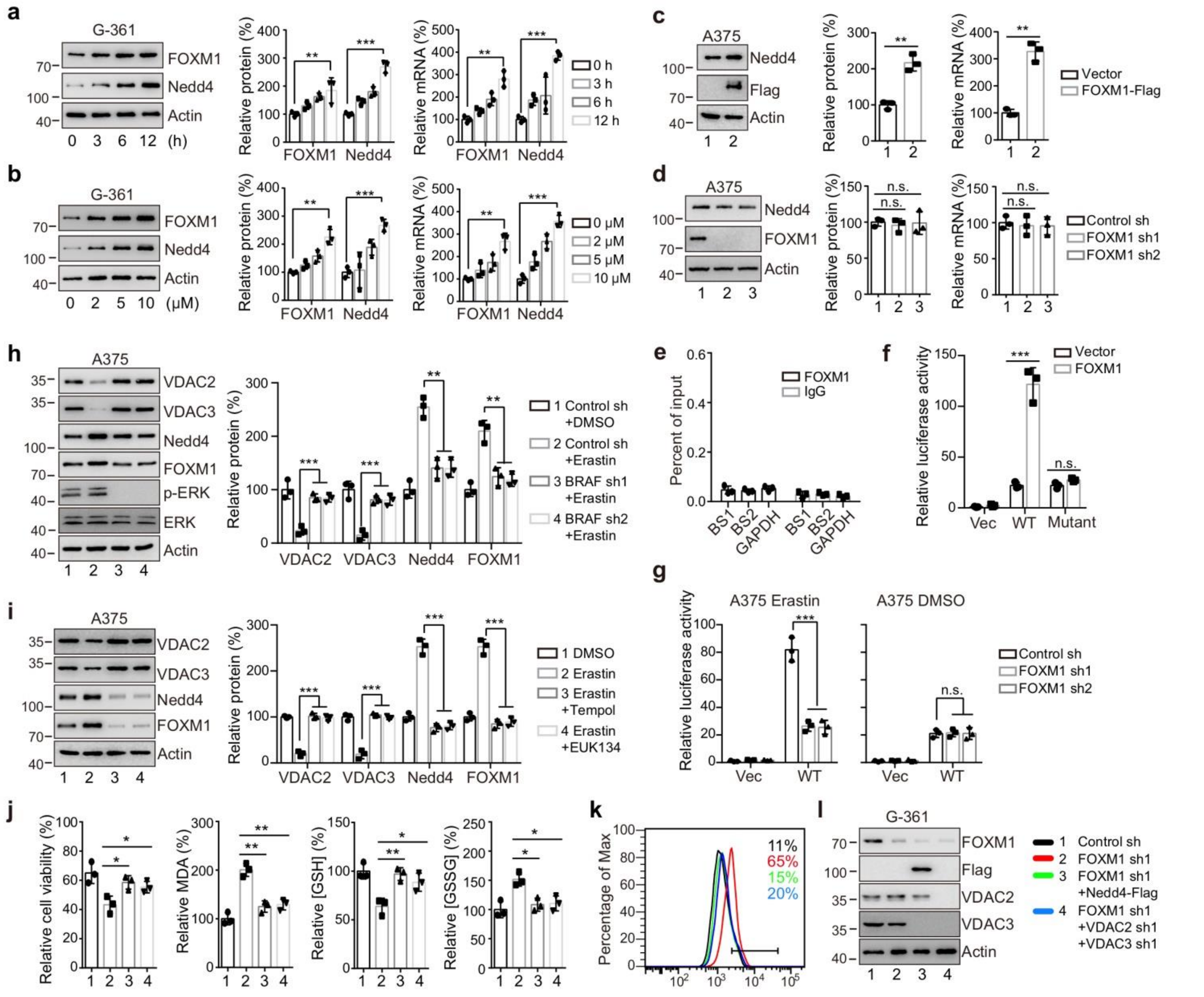

Supplementary Figure 9. Upregulation of Nedd4 by erastin was mediated by FOXM1

(**a, b**) Erastin induced the expression of FOXM1 and Nedd4 in G-361 cells. Cells were treated with erastin at different time points (0-12 h) or different concentrations (0-10 μM). Cell lysates were analyzed by immunoblotting with indicated antibodies. The protein levels of FOXM1 and Nedd4 were quantitated by Image J and normalized to actin levels. (**c**) FOXM1 induces the expression of Nedd4. The protein and mRNA levels of Nedd4 were detected in A375 cells expressing FOXM1 or control vector. (**d**) Knockdown of FOXM1 cannot influence the expression of Nedd4. The protein and mRNA levels of Nedd4 were detected in A375 cells expressing FOXM1 shRNA or control shRNA vectors. (**e**) ChIP analysis shows no occupancy by FOXM1 on *Nedd4* promoter in A375 without erastin treatment. GAPDH promoter serves as a negative control. (**f**) Dual-luciferase reporter assay showing the activity of *Nedd4* promoter in response to FOXM1 overexpression in A375 cells. The *Nedd4* promoter-reporters were transfected into indicated A375 cells for 24 h, and then luciferase activity

was measured. (**g**) Dual-luciferase reporter assay showing the activity of *Nedd4* promoter in response to erastin was mediated by FOXM1. The *Nedd4* promoter-reporters were transfected into indicated A375 cells for 24 h, and cells were treated by erastin (5 μM) or DMSO for an additional 12 h, then luciferase activity was measured. (**h**) Knockdown of BRAF suppressed erastin-induced FOXM1 and Nedd4 expression. A375 cells expressing control shRNA or BRAF shRNA constructs were treated with DMSO or erastin (5 μM) for 12 h, the protein levels of VDAC2, VDAC3, Nedd4, and FOXM1 were assayed by western blot. (**i**) ROS is essential for the upregulation of FOXM1 and Nedd4 induced by erastin. A375 cells were treated with indicated chemicals (erastin 5 μM, Tempol 10 μM, EUK134 5 μM) for 12 h, the protein levels of VDAC2, VDAC3, Nedd4, and FOXM1 were assayed by western blot. (**j-l**) Overexpression of Nedd4 or knockdown of VDAC2/3 suppressed erastin-induced ferroptotic cell death which was enhanced by FOXM1 knockdown. G-361 cells transfected with indicated DNA constructs for 48 h, and treated with erastin (5 μM) for 12 h. Cell viability, intracellular MDA, GSH, and GSSG levels were measured (j). The lipid ROS level was assessed by flow cytometry using C11-BODIPY (k). The protein levels of overexpressed constructs were assessed by immunoblotting (l). Data shown represent as mean ± SD from three independent experiments. Comparisons were made using Student's t-test. *, $p < 0.05$; **, $p < 0.01$; ***, $p < 0.001$; n.s., not significant.

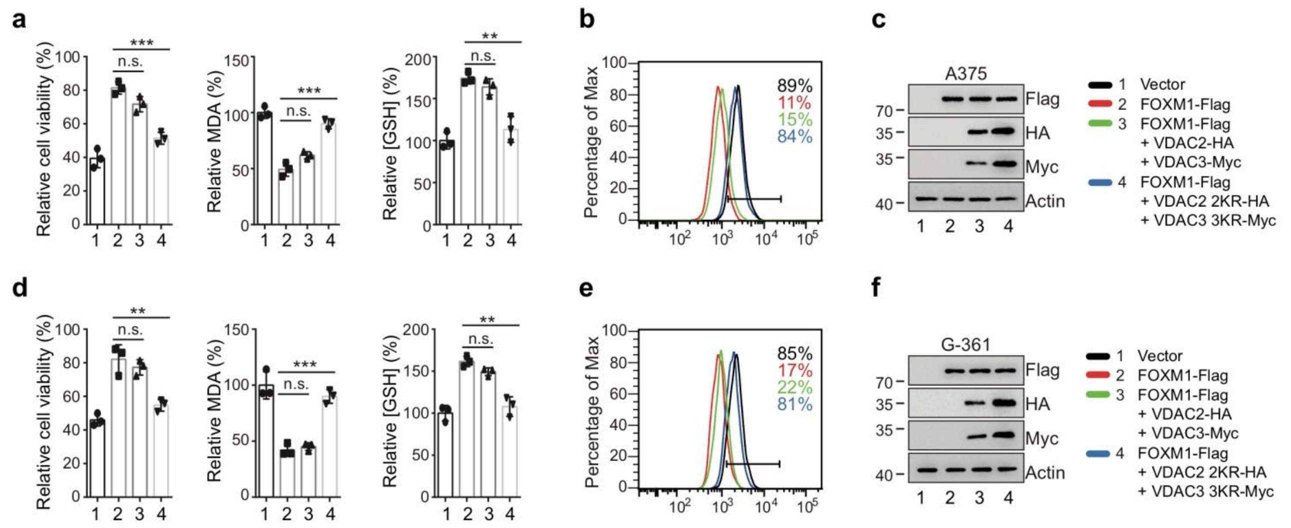

Supplementary Figure 10. Overexpression of KR mutants of VDAC2/3 increased erastin-induced ferroptosis which was inhibited by FOXM1.

(**a-c**) A375 cells were transfected with indicated DNA constructs for 48 h, and treated with erastin (5 μM) for 12 h. Cell viability, intracellular MDA, and GSH levels were measured (a). The lipid ROS level was assessed by flow cytometry using C11-BODIPY (b). The protein levels of overexpressed constructs were assessed by immunoblotting (c). (**d-f**) G-361 cells were transfected with indicated DNA constructs for 48 h, and treated with erastin (10 μM) for 12 h. Cell viability, intracellular MDA, and GSH levels were measured (d). The lipid ROS level was assessed by flow cytometry using C11-BODIPY (e). The protein levels of overexpressed constructs were assessed by immunoblotting (f). Data shown represent as mean ± SD from three independent experiments. Comparisons were made using Student's t-test. **, $p < 0.01$; ***, $p < 0.001$; n.s., not significant.

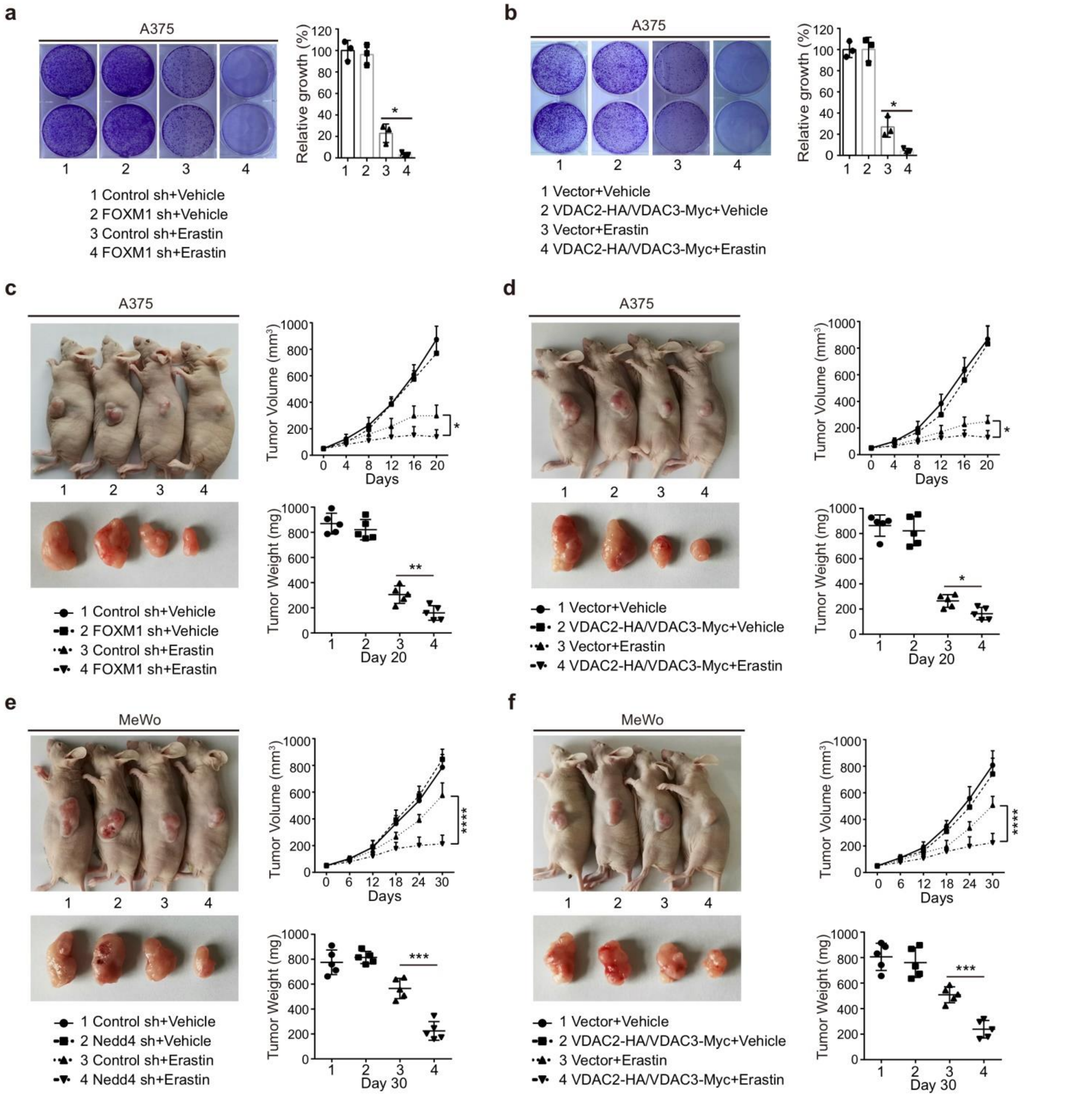

Supplementary Figure 11. FOXM1 and VDAC2/3 regulate the anti-tumor activity of erastin. (**a, b**) Colony formation assay of indicated A375 cells. Cells were treated with erastin (5 µM) for 24 h and were grown without erastin for 10 days. For each cell line, all dishes were fixed, stained, and photographed at the same time. Data are mean ± SD from three independent experiments. (**c, d**) Knockdown of FOXM1 (c) or overexpression of VDAC2/3 (d) enhanced erastin-induced ferroptosis *in vivo*. The 7-week-old immunodeficient nude mice were injected subcutaneously with indicated A375 cells (5X10^6 cells/mouse) and treated with erastin (15 mg/kg intraperitoneal, twice every other day) when the tumor volume reached 50 mm^3. Tumor volume was calculated every four days, and the tumor mess was measured at day 20. (**e, f**) Knockdown of Nedd4 (e) or overexpression of VDAC2/3 (f) enhances erastin-induced ferroptosis *in vivo*. The 7-week-old immunodeficient nude mice were

injected subcutaneously with indicated MeWo cells ($5X10^6$ cells/mouse) and treated with erastin (15 mg/kg intraperitoneal, twice every other day) when the tumor volume reached 50 mm^3. Tumor volume was calculated every six days (e), and the tumor mess was measured at day 30 (f). Data represents mean ± SD (n = 5 mice/group). Comparisons were made using Student's t-test. *, $p < 0.05$; **, $p < 0.01$; ***, $p < 0.001$; ****, $p < 0.0001$.

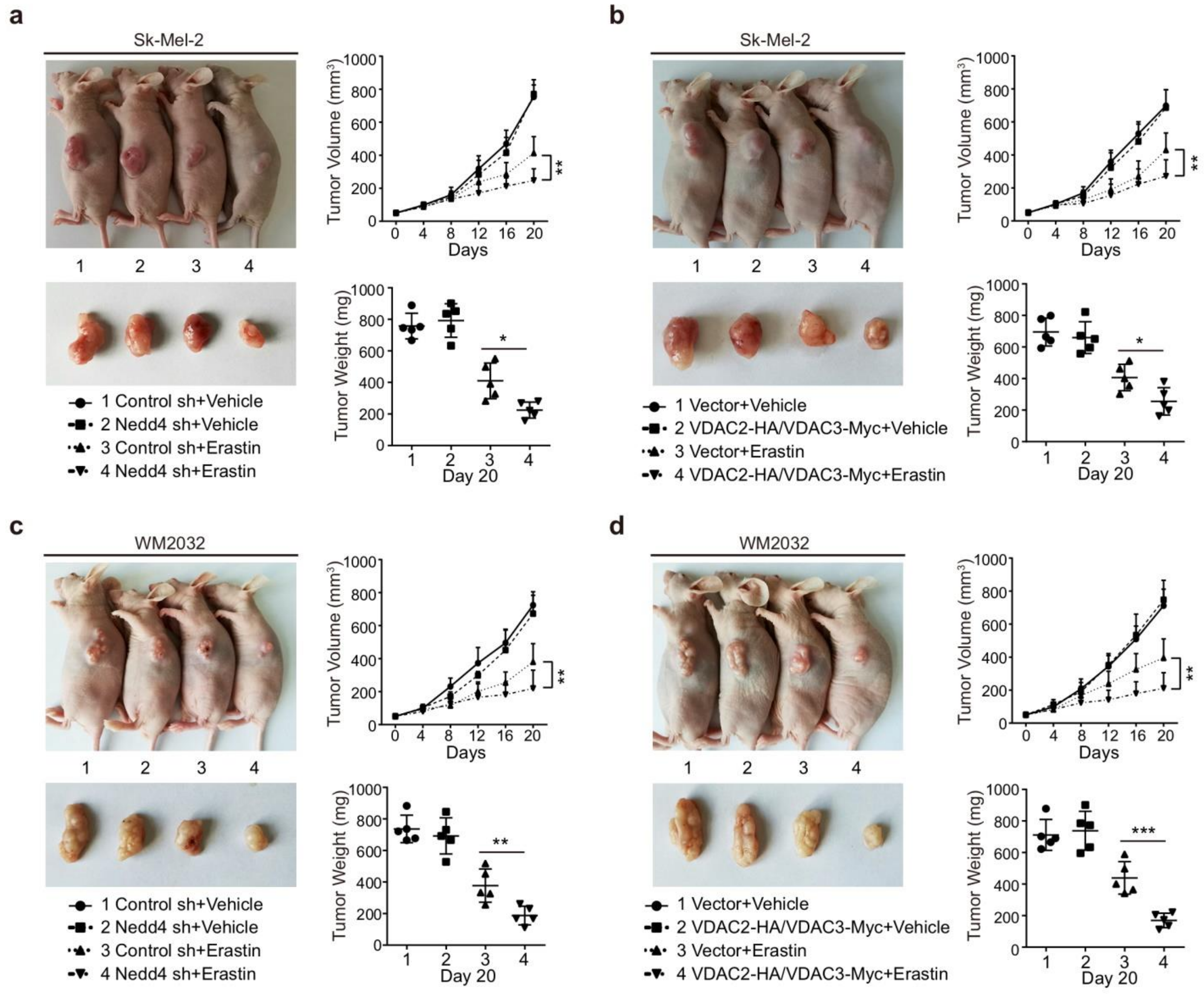

Supplementary Figure 12. Nedd4 and VDAC2/3 regulate the anti-tumor activity of erastin in NRAS-mutant melanoma cells.

(**a, b**) Knockdown of Nedd4 (a) or overexpression of VDAC2/3 (b) enhanced erastin-induced ferroptosis in SK-MEL-2 cells. The 7-week-old immunodeficient nude mice were injected subcutaneously with SK-MEL-2 cells ($5X10^6$ cells/mouse) and treated with erastin (15 mg/kg intraperitoneal, twice every other day) when the tumor volume reached 50 mm^3. Tumor volume was calculated every four days, and the tumor mess was measured at day 20. (**c, d**) Knockdown of Nedd4 (c) or overexpression of VDAC2/3 (d) enhanced erastin-induced ferroptosis in WM2032 cells. The 7-week-old immunodeficient nude mice were injected subcutaneously with indicated WM2032 cells ($5X10^6$ cells/mouse) and treated with erastin (15 mg/kg intraperitoneal, twice every other day) when the tumor volume reached 50 mm^3. Tumor volume was calculated every four days (c), and the tumor mess was measured at day 20 (d). Data represents mean ± SD (n = 5 mice/group). Comparisons were made using Student's t-test. *, $p < 0.05$; **, $p < 0.01$; ***, $p < 0.001$.

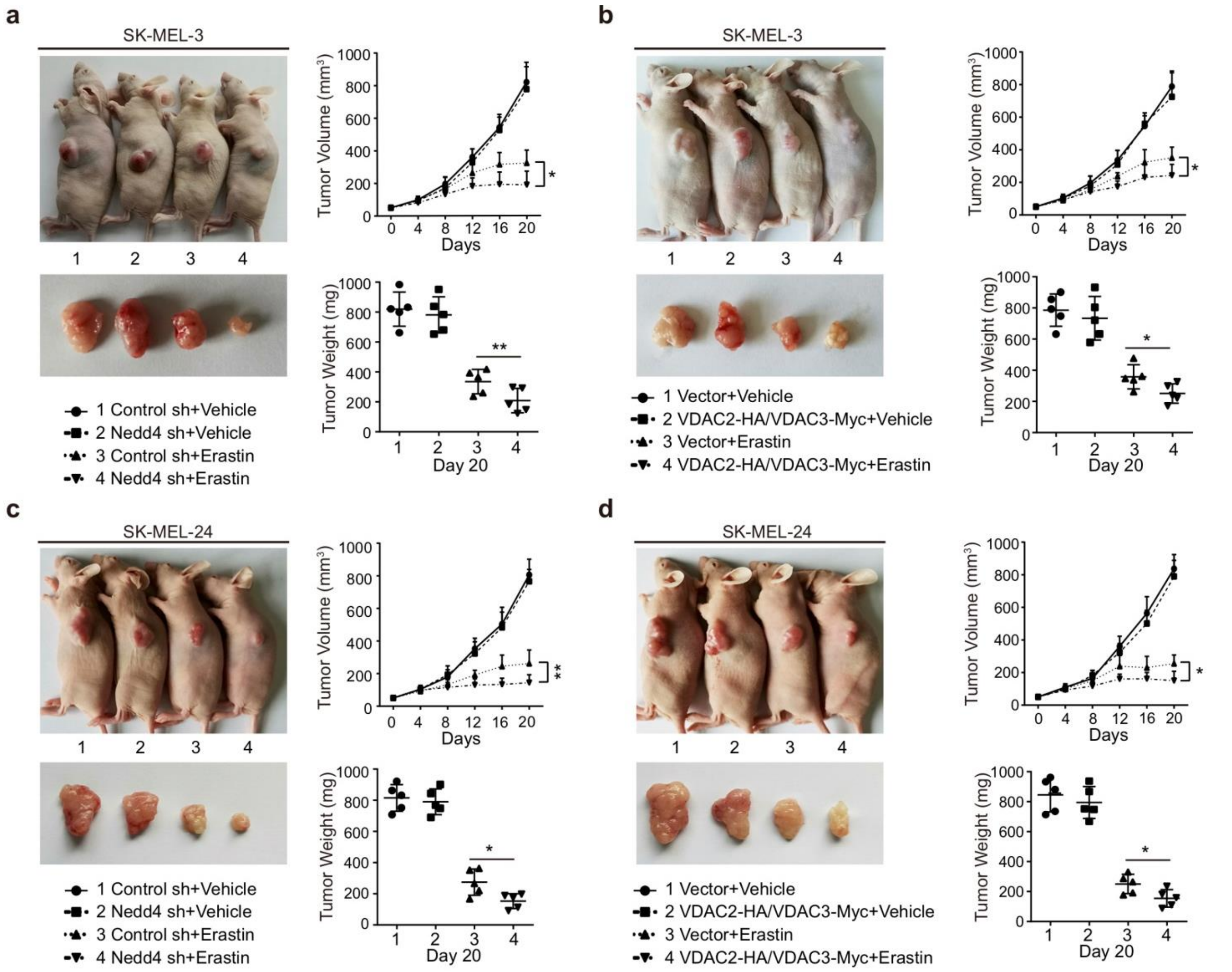

Supplementary Figure 13. Nedd4 and VDAC2/3 regulate the anti-tumor activity of erastin in PTEN-mutant melanoma cells.

(**a, b**) Knockdown of Nedd4 (a) or overexpression of VDAC2/3 (b) enhanced erastin-induced ferroptosis in SK-MEL-3 cells. The 7-week-old immunodeficient nude mice were injected subcutaneously with SK-MEL-3 cells ($5X10^6$ cells/mouse) and treated with erastin (15 mg/kg intraperitoneal, twice every other day) when the tumor volume reached 50 mm^3. Tumor volume was calculated every four days (a), and the tumor mess was measured at day 20 (b). (**c, d**) Knockdown of Nedd4 (c) or overexpression of VDAC2/3 (d) enhanced erastin-induced ferroptosis in SK-MEL-24 cells. The 7-week-old immunodeficient nude mice were injected subcutaneously with SK-MEL-24 cells ($5X10^6$ cells/mouse) and treated with erastin (15 mg/kg intraperitoneal, twice every other day) when the tumor volume reached 50 mm^3. Tumor volume was calculated every four days (c), and the tumor mess was measured at day 20 (d). Data represents mean ± SD (n = 5 mice/group). Comparisons were made using Student's t-test. *, $p < 0.05$; **, $p < 0.01$.

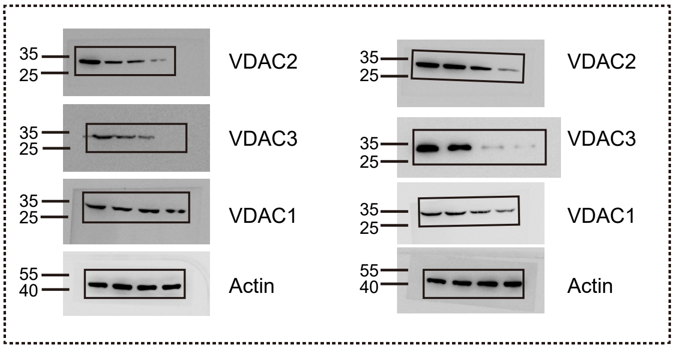

Figure 1b

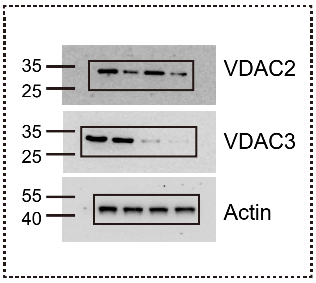

Figure 1d

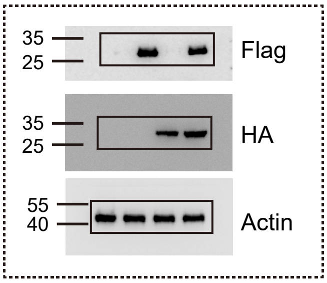

Figure 1f

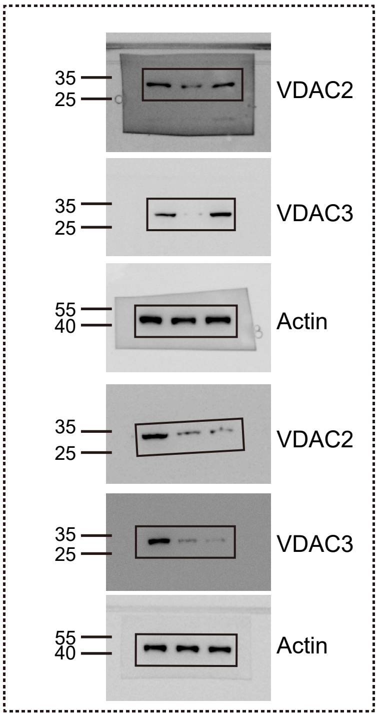

Figure 1g

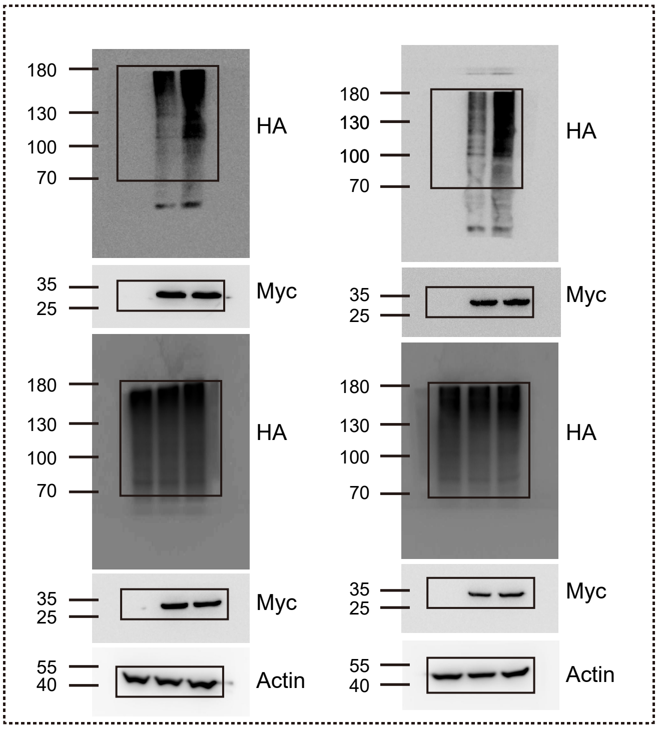

Figure 1h

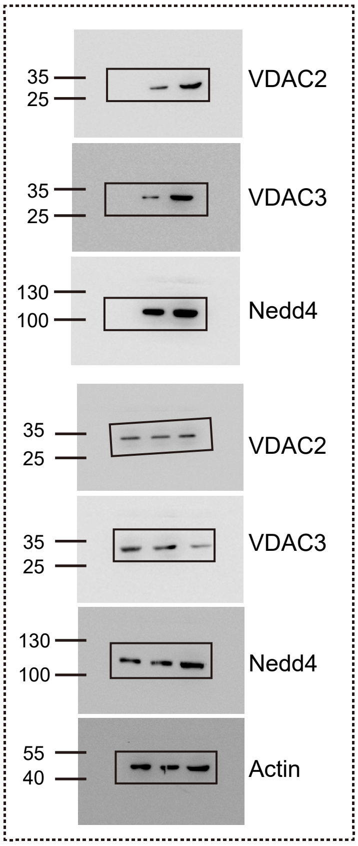

Figure 2b

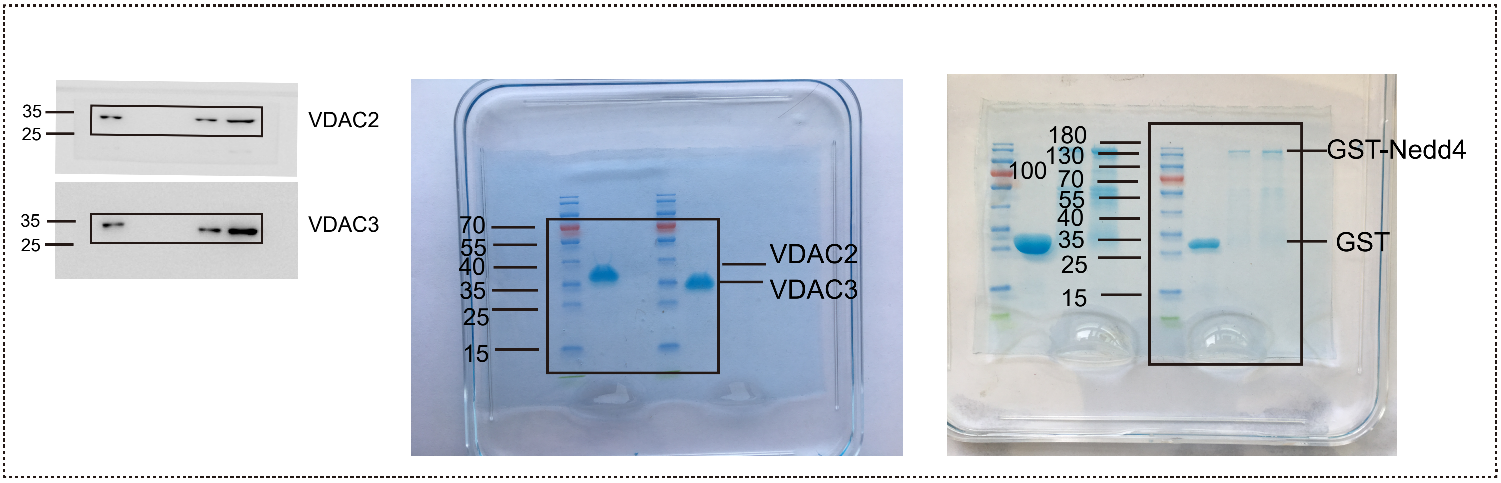

Figure 2c

Supplementary Figure 14: uncropped scans with size marker indication

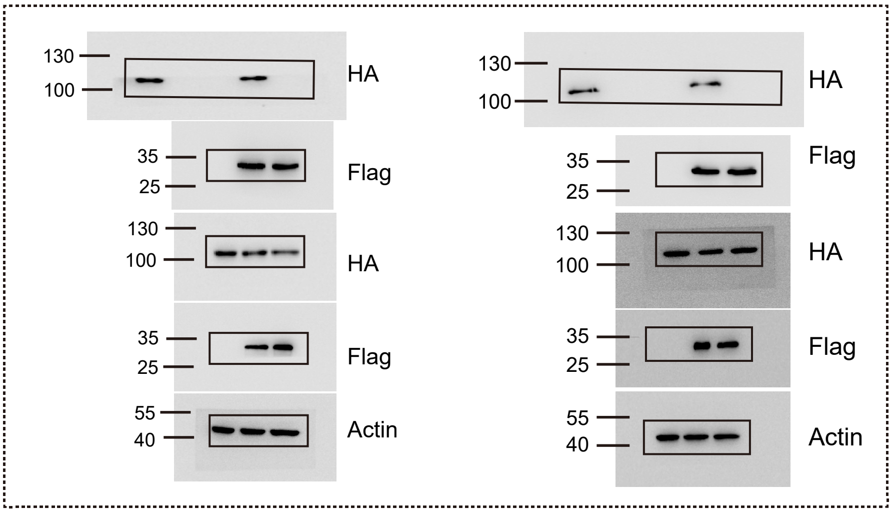

Figure 2d

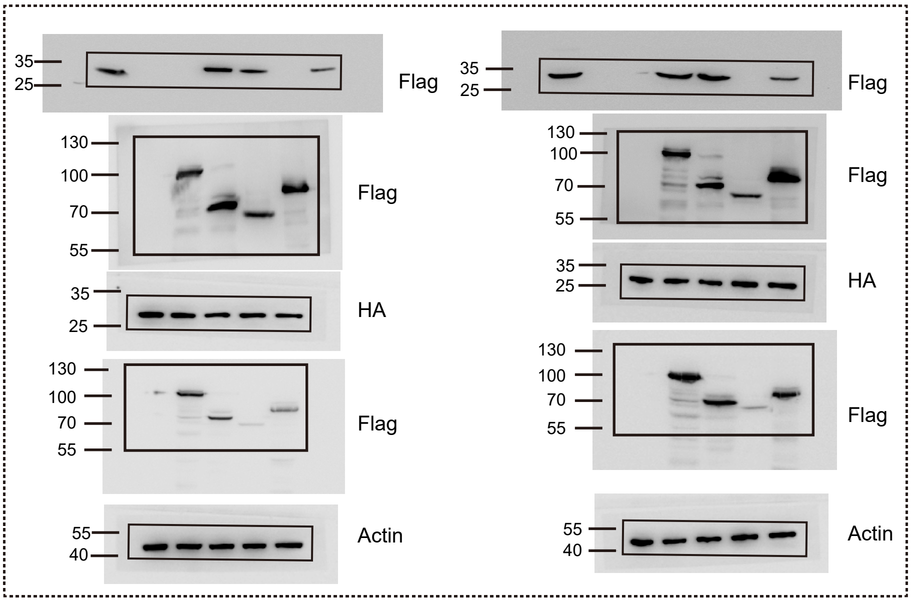

Figure 2f

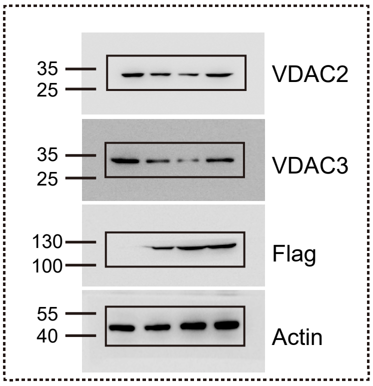

Figure 3a

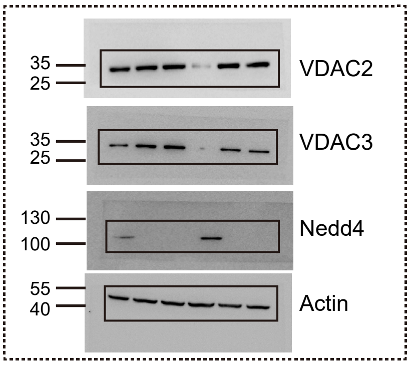

Figure 3b

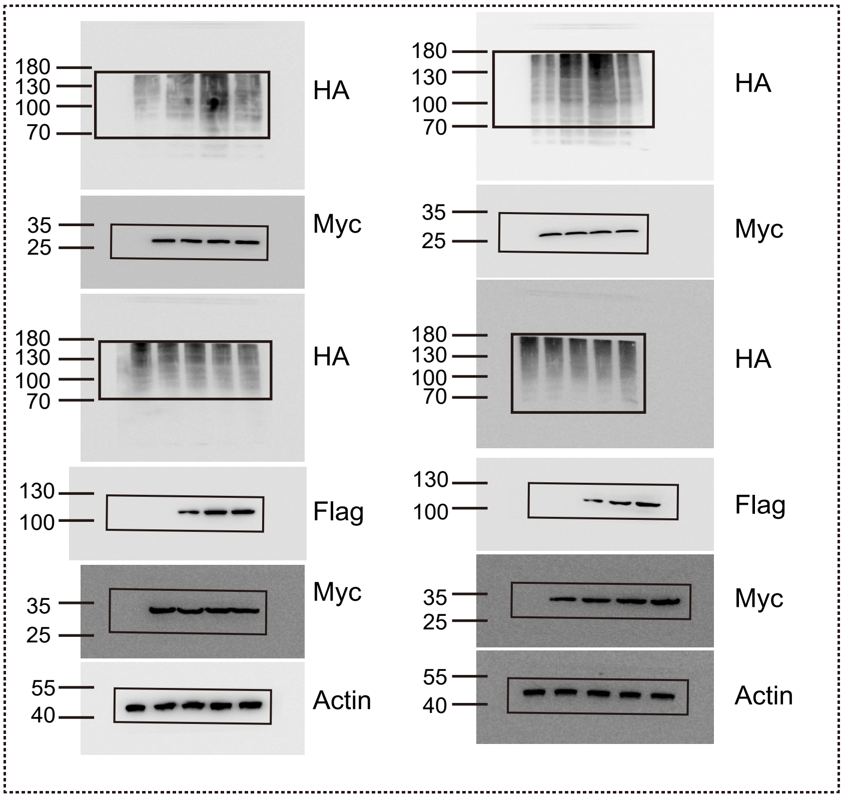

Figure 3c

Supplementary Figure 14 continued

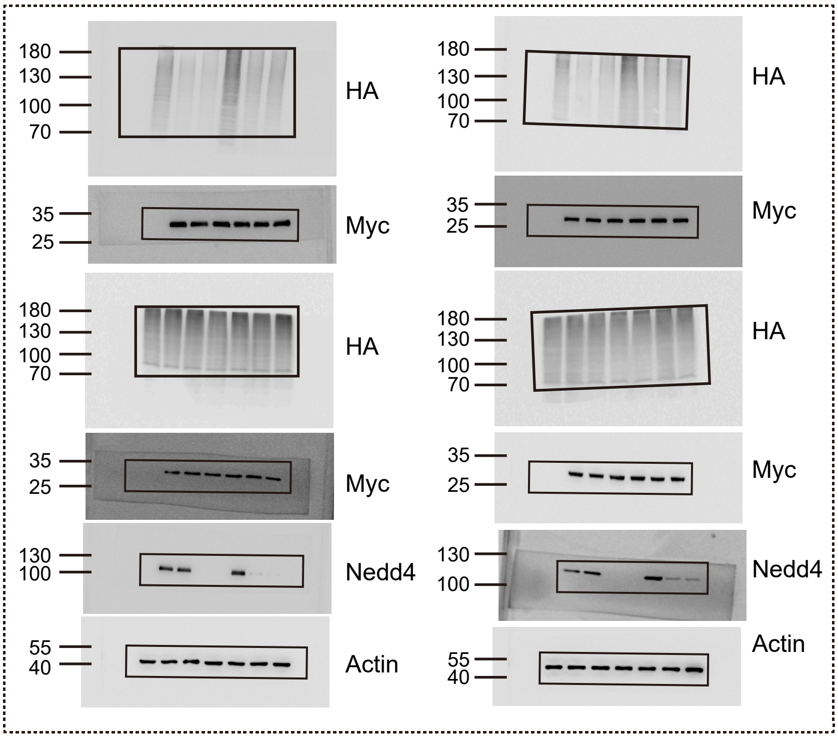

Figure 3d

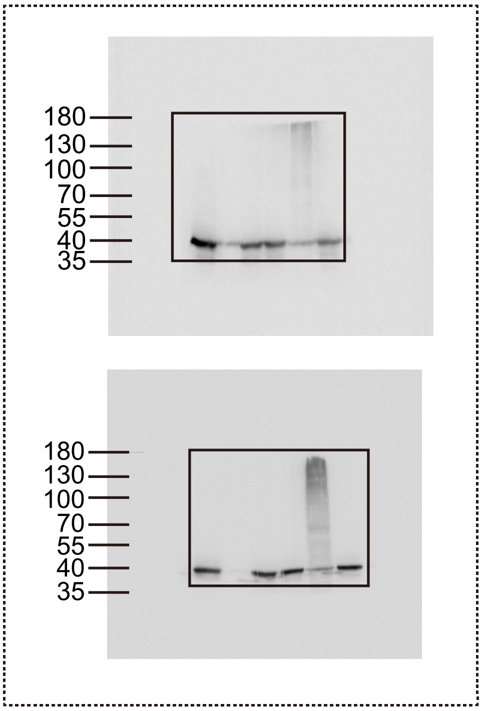

Figure 3e

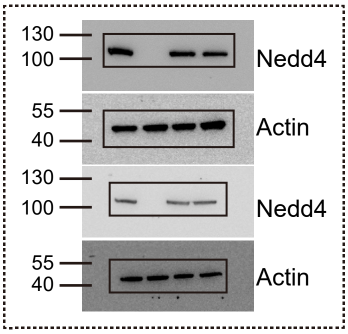

Figure 4f

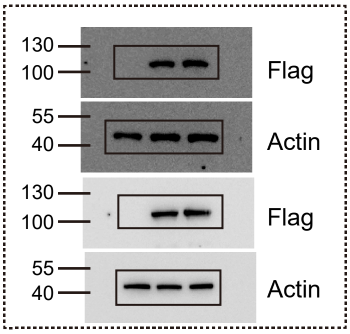

Figure 4i

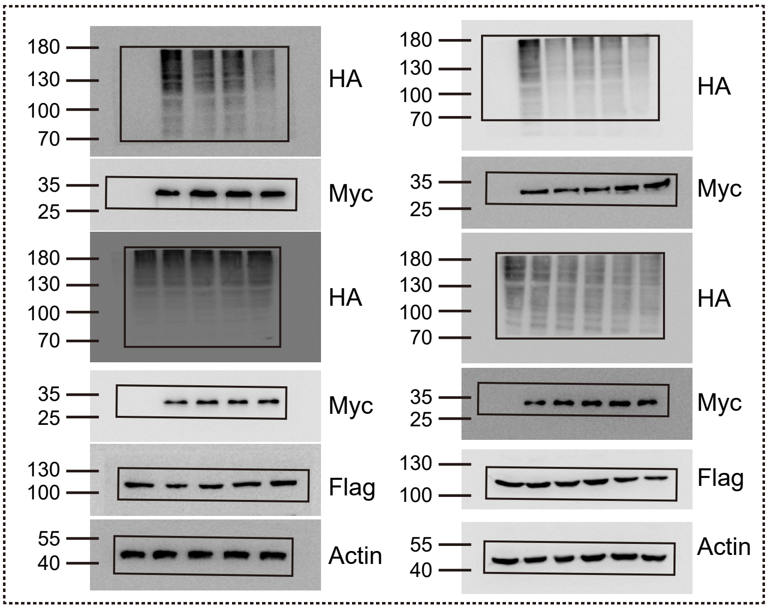

Figure 5a

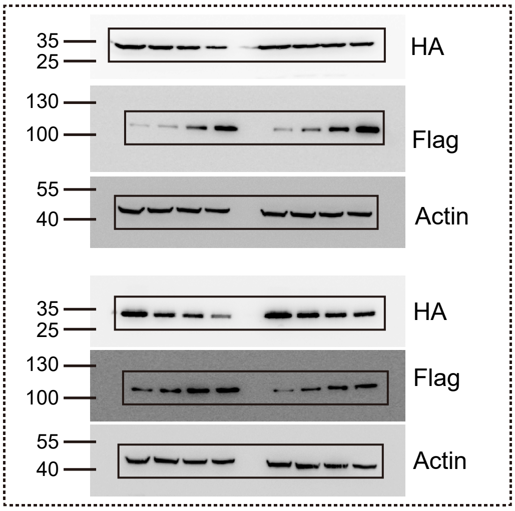

Figure 5b

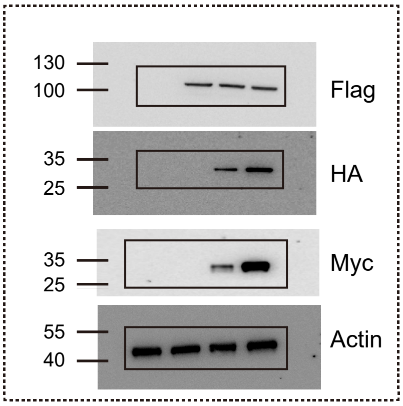

Figure 5e

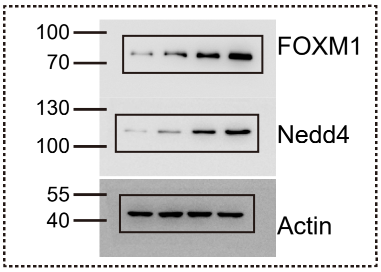

Figure 6a

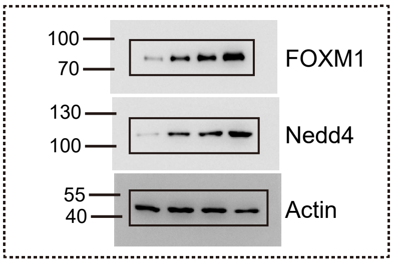

Figure 6b

Supplementary Figure 14 continued

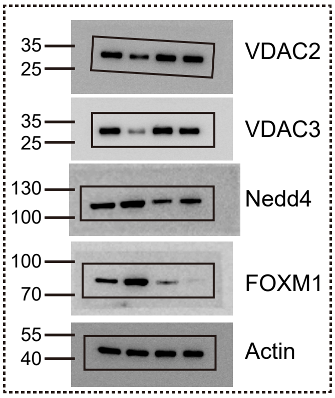

Figure 6f

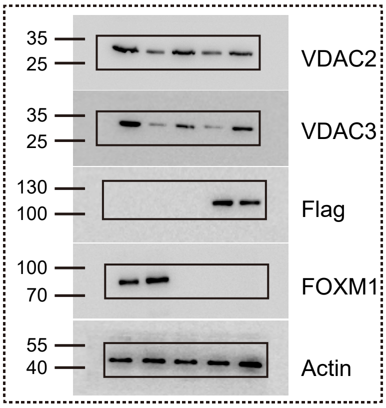

Figure 6g

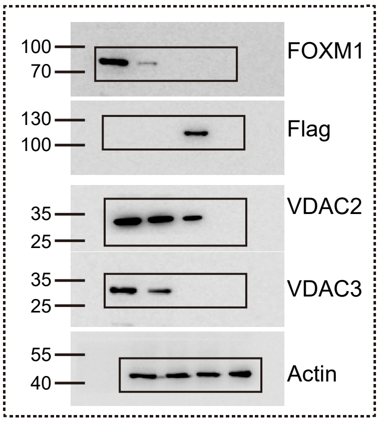

Figure 6j

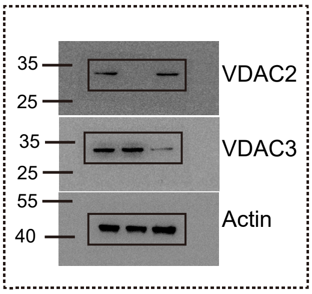

Supplementary Figure 1a

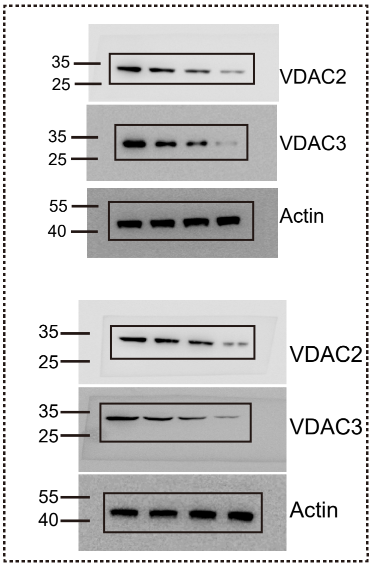

Supplementary Figure 1b

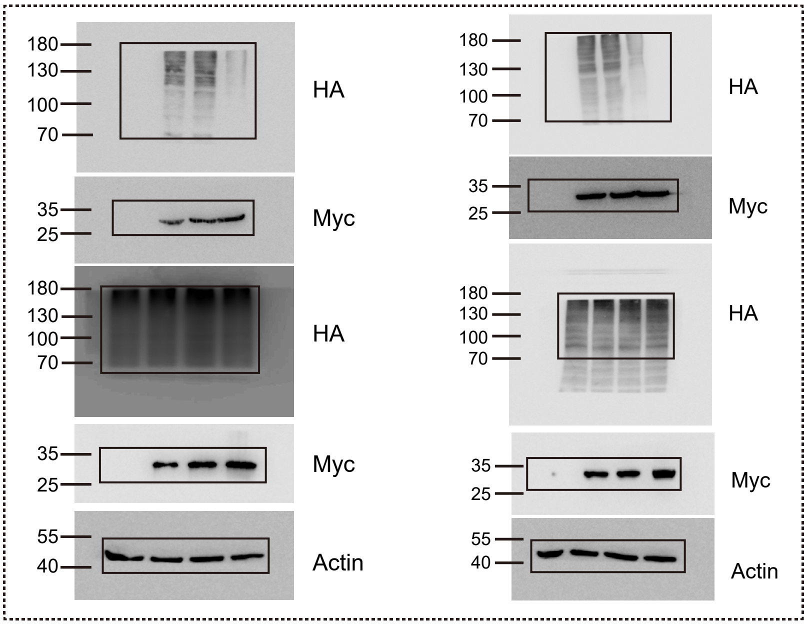

Supplementary Figure 1d

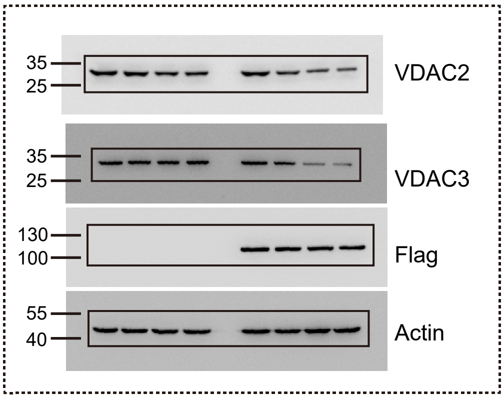

Supplementary Figure 3a

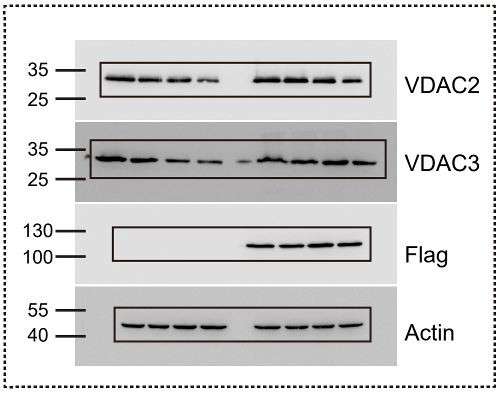

Supplementary Figure 3b

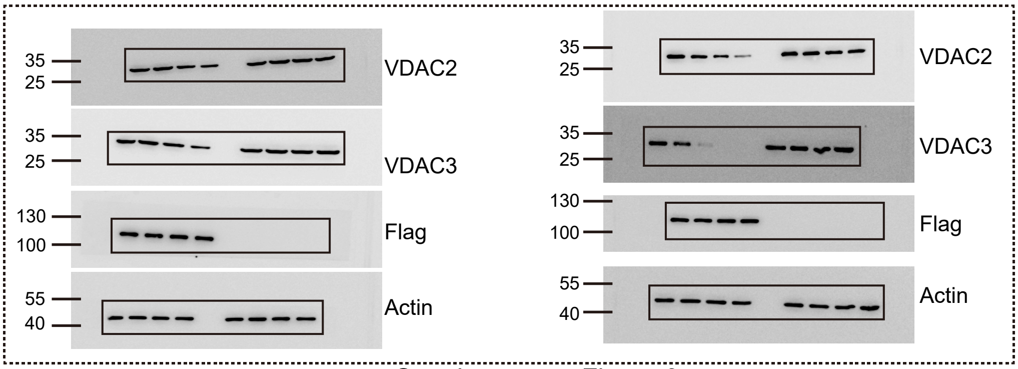

Supplementary Figure 3c

Supplementary Figure 14 continued

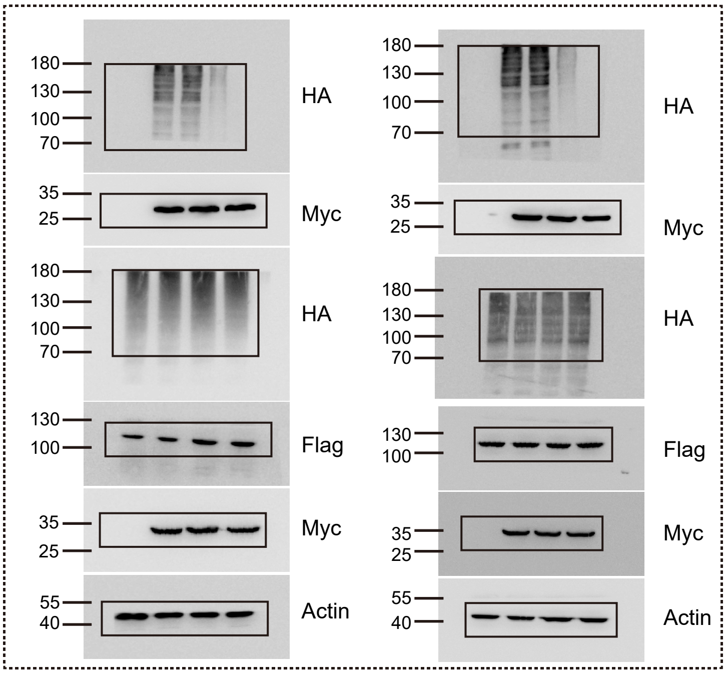

Supplementary Figure 3d

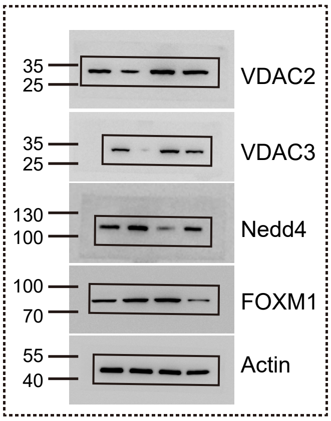

Supplementary Figure 5a

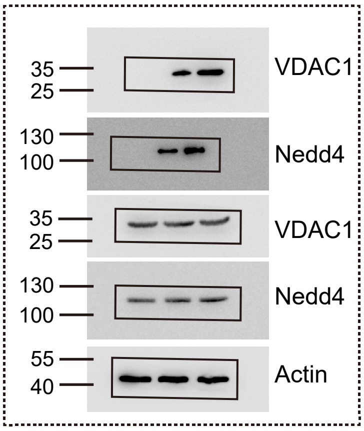

Supplementary Figure 6c

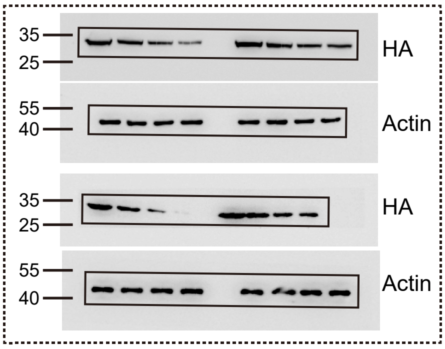

Supplementary Figure 7a

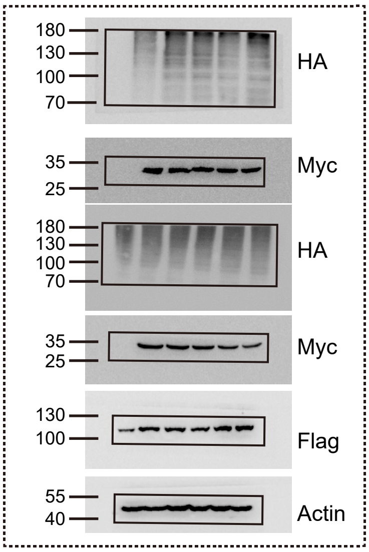

Supplementary Figure 7b

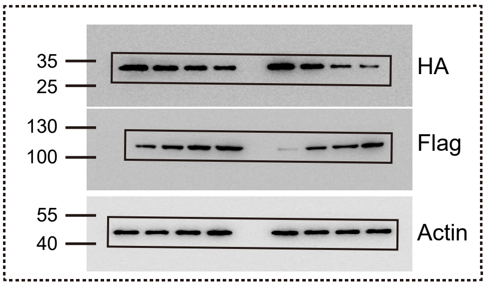

Supplementary Figure 7c

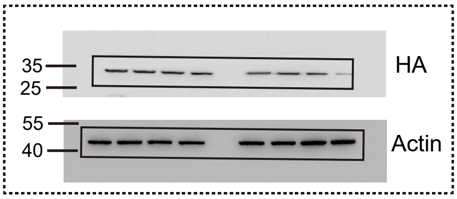

Supplementary Figure 7d

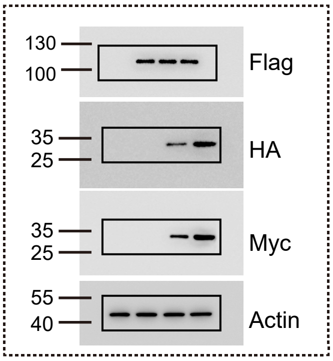

Supplementary Figure 8c

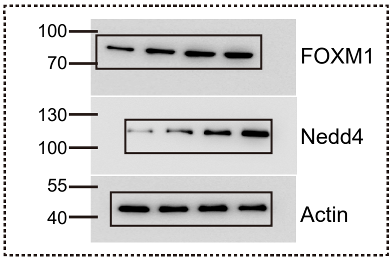

Supplementary Figure 9a

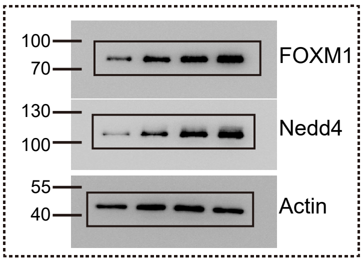

Supplementary Figure 9b

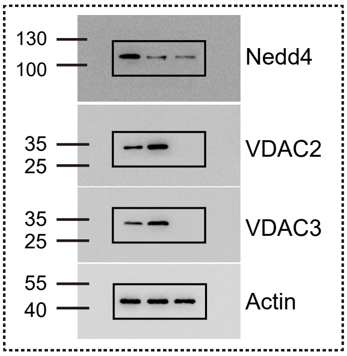

Supplementary Figure 8f

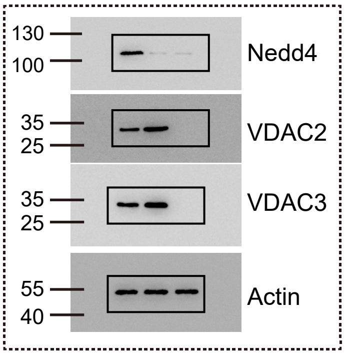

Supplementary Figure 8i

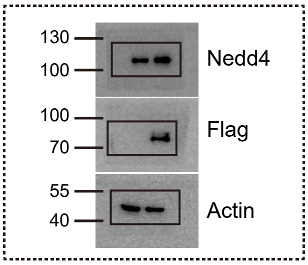

Supplementary Figure 9c

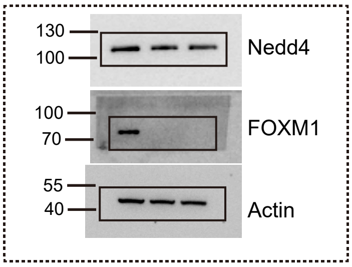

Supplementary Figure 9d

Supplementary Figure 14 continued

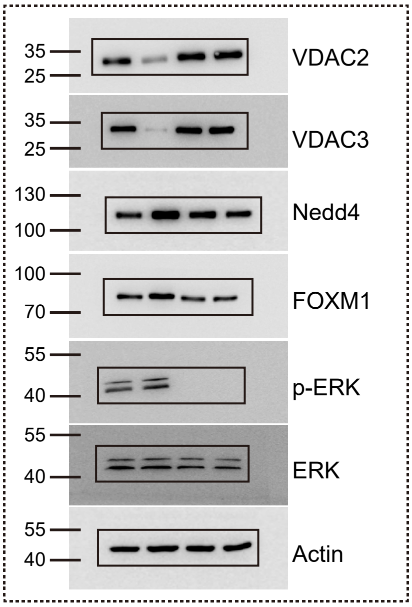

Supplementary Figure 9h

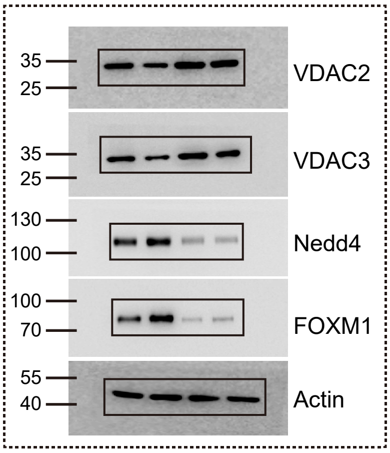

Supplementary Figure 9i

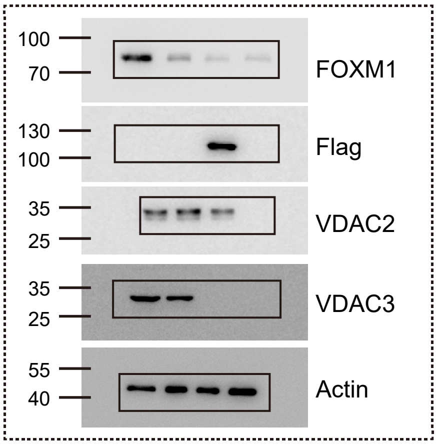

Supplementary Figure 9l

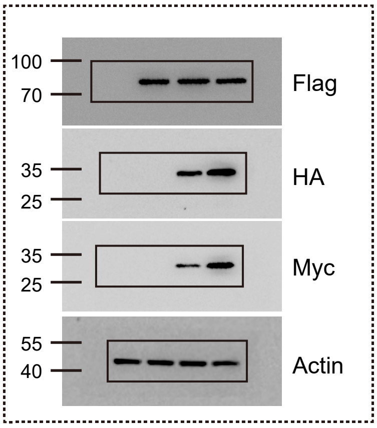

Supplementary Figure 10c

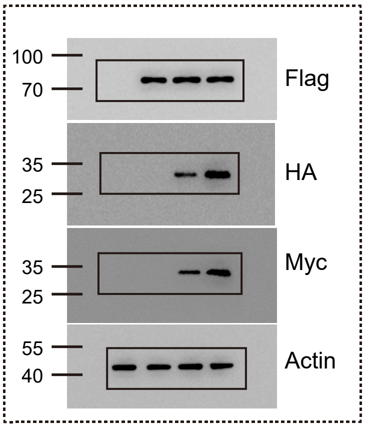

Supplementary Figure 10f

Supplementary Figure 14 continued

Supplementary References:

1. **Li Y*, et al.* An integrated bioinformatics platform for investigating the human E3 ubiquitin ligase-substrate interaction network. *Nat Commun* 8, 347 (2017).**
