# Supplementary material for: Nedd4 ubiquitylates VDAC2/3 to suppress erastin-induced ferroptosis in melanoma
Source: Nat Commun. 2020 Jan 23;11:433. doi: 10.1038/s41467-020-14324-x (PMC6978386; doi:10.1038/s41467-020-14324-x)
Supplement: Supplementary file 3 — Reporting Summary [file 41467_2020_14324_MOESM3_ESM.pdf]

nature research

Corresponding author(s): Dr. Yongfei Yang

Last updated by author(s): Nov 20, 2019

Reporting Summary

Nature Research wishes to improve the reproducibility of the work that we publish. This form provides structure for consistency and transparency in reporting. For further information on Nature Research policies, seeAuthors & Referees and theEditorial Policy Checklist .

Statistics

For all statistical analyses, confirm that the following items are present in the figure legend, table legend, main text, or Methods section.

n/a	Confirmed	
☐	☒	The exact sample size (*n*) for each experimental group/condition, given as a discrete number and unit of measurement
☐	☒	A statement on whether measurements were taken from distinct samples or whether the same sample was measured repeatedly
☐	☒	The statistical test(s) used AND whether they are one- or two-sided<br>*Only common tests should be described solely by name; describe more complex techniques in the Methods section.*
☒	☐	A description of all covariates tested
☒	☐	A description of any assumptions or corrections, such as tests of normality and adjustment for multiple comparisons
☐	☒	A full description of the statistical parameters including central tendency (e.g. means) or other basic estimates (e.g. regression coefficient) AND variation (e.g. standard deviation) or associated estimates of uncertainty (e.g. confidence intervals)
☐	☒	For null hypothesis testing, the test statistic (e.g. *F*, *t*, *r*) with confidence intervals, effect sizes, degrees of freedom and *P* value noted<br>*Give P values as exact values whenever suitable.*
☒	☐	For Bayesian analysis, information on the choice of priors and Markov chain Monte Carlo settings
☒	☐	For hierarchical and complex designs, identification of the appropriate level for tests and full reporting of outcomes
☒	☐	Estimates of effect sizes (e.g. Cohen's *d*, Pearson's *r*), indicating how they were calculated

Our web collection on statistics for biologists contains articles on many of the points above.

Software and code

Policy information about availability of computer code

Data collection	Zen (Zeiss) software was used to collect confocal images; BD FACSDiva (BD Biosciences) was used to collect flow cytometry data;
Data analysis	ImageJ were used for confocal images analysis; Graphpad were used for bar graphs output and statistical analysis; FlowJo_V7.6 was used for flow data analysis. The software and algorithms for data analyses used in this study are all well-established from previous work. All software and custom arguments are included in Methods section. There is no unreported algorithm used in this paper. The source codes for data processing are available from the corresponding author on reasonable request.

For manuscripts utilizing custom algorithms or software that are central to the research but not yet described in published literature, software must be made available to editors/reviewers. We strongly encourage code deposition in a community repository (e.g. GitHub). See the Nature Research guidelines for submitting code & software for further information.

Data

Policy information about availability of data

All manuscripts must include a data availability statement. This statement should provide the following information, where applicable:
- Accession codes, unique identifiers, or web links for publicly available datasets
- A list of figures that have associated raw data
- A description of any restrictions on data availability

UbiBrowser (http://ubibrowser.ncpsb.org/) was used to predict the E3 ligases of VDAC isoforms. All data supporting the findings of this study are available from the corresponding author on reasonable request.

Field-specific reporting

Please select the one below that is the best fit for your research. If you are not sure, read the appropriate sections before making your selection.

- [x] Life sciences
- [ ] Behavioural & social sciences
- [ ] Ecological, evolutionary & environmental sciences

For a reference copy of the document with all sections, see nature.com/documents/nr-reporting-summary-flat.pdf

Life sciences study design

All studies must disclose on these points even when the disclosure is negative.

Sample size	No sample size calculations were performed. Sample size was determined according to our experience as well as literature reporting in terms of specific experiment.
Data exclusions	No samples or animals were excluded from the analyses.
Replication	Multiple independent repeats were included for related experiments. Each experiment was performed for at least three times to make sure similar results are reproducible.
Randomization	6-8 week female nude mice were chosen as xenograft hosts, and randomly allocated into experimental groups.
Blinding	For cell-based experiments, cell types were known when prepare the samples or start to treat cells at the beginning of experiments, data collection were blinded to different person who processed assay at the time.

Reporting for specific materials, systems and methods

We require information from authors about some types of materials, experimental systems and methods used in many studies. Here, indicate whether each material, system or method listed is relevant to your study. If you are not sure if a list item applies to your research, read the appropriate section before selecting a response.

Materials & experimental systems

n/a	Involved in the study	
☐	☒	Antibodies
☐	☒	Eukaryotic cell lines
☒	☐	Palaeontology
☐	☒	Animals and other organisms
☒	☐	Human research participants
☒	☐	Clinical data

Methods

n/a	Involved in the study	
☒	☐	ChIP-seq
☐	☒	Flow cytometry
☒	☐	MRI-based neuroimaging

Antibodies

Antibodies used	The following antibodies were used in this study: Nedd4 (PA5-17463, Thermo), VDAC1 (MABN504,Merck), VDAC2 (Abcam, ab37985), VDAC3 (Abcam, ab130561), FOXM1 (702664, Thermo), 4HNE (Abcam, ab46545), Flag (F3165, clone M2; Sigma), HA (H6533, Sigma), HA (PRB-101P, Covance), GST (PA1982A, Thermo), Myc (AH00052, Thermo), Actin (PA116889, Thermo), p44/42 (4695,Cell Signaling), Phospho-p44/42 (4370, Cell Signaling).
Validation	All antibodies used in our study have been validated and detailed information could be found on the website from manufactures as listed below. Some of them have also been validated by our experiments as shown in this manuscript using either overexpress,<br>knockout or knockdown strategies.<br>Nedd4, https://www.thermofisher.com/antibody/product/NEDD4-Antibody-Polyclonal/PA5-17463;<br>VDAC1, http://www.yl-guolv.com/millipore/mabn504.html;<br>VDAC2, https://www.abcam.com/vdac2-antibody-ab37985.html;<br>VDAC3, https://www.abcam.com/vdac3-antibody-ab130561.html;<br>FOXM1, https://www.thermofisher.com/antibody/product/FOXM1-Antibody-clone-1H24L2-Monoclonal/702664;<br>4HNE, https://www.abcam.com/4-Hydroxynonenal-antibody-ab46545.html<br>Flag, https://www.sigmaaldrich.com/catalog/product/sigma/f3165?lang=en&region=US;<br>HA, https://www.sigmaaldrich.com/catalog/product/sigma/h6533?lang=en&region=US;<br>HA, https://www.biolegend.com/en-us/products/purified-anti-ha-11-epitope-tag-antibody-11375<br>GST, https://www.thermofisher.com/antibody/product/GST-Tag-Antibody-Polyclonal/PA1-982A;<br>Myc, https://www.thermofisher.com/antibody/product/c-Myc-Antibody-clone-9E10-Monoclonal/MA1-980;<br>Actin, https://www.thermofisher.com/antibody/product/beta-Actin-Antibody-Polyclonal/PA1-16889;

Erk, https://www.cellsignal.com/products/primary-antibodies/p44-42-mapk-erk1-2-137f5-rabbit-mab/4695;
Phospho-Erk, https://www.cellsignal.com/products/primary-antibodies/phospho-p44-42-mapk-erk1-2-thr202-tyr204-d13-14-4e-xp-rabbit-mab/4370.

Eukaryotic cell lines

Policy information about cell lines

Cell line source(s)	A375, G-361, MeWo, SK-MEL-2, SK-MEL-3, SK-MEL-24 , WM2032 and HEK293T cell lines were obtained from American Type Culture Collection (ATCC).
Authentication	Cell lines were not authenticated.
Mycoplasma contamination	All cell lines are tested negative for mycoplasma contamination
Commonly misidentified lines (See ICLAC register)	HEK-293T cells were used to for lentiviral production.

Animals and other organisms

Policy information about studies involving animals; ARRIVE guidelines recommended for reporting animal research

Laboratory animals	NU/J nude female mice at 7-week-old were purchased from Beijing Vital River Laboratory Animal Technology Co., Ltd., For Tumor xenograft models, tumor cells were were injected subcutaneously into the right posterior flanks of 7-week-old female nude mice. All animal experiments were approved by Beijing Institute of Biotechnology.
Wild animals	No wild animals involved in this study.
Field-collected samples	This study didn't involve samples collected from field.
Ethics oversight	Beijing Institute of Biotechnology

Note that full information on the approval of the study protocol must also be provided in the manuscript.

Flow Cytometry

Plots

Confirm that:

- [x] The axis labels state the marker and fluorochrome used (e.g. CD4-FITC).
- [x] The axis scales are clearly visible. Include numbers along axes only for bottom left plot of group (a 'group' is an analysis of identical markers).
- [x] All plots are contour plots with outliers or pseudocolor plots.
- [x] A numerical value for number of cells or percentage (with statistics) is provided.

Methodology

Sample preparation	Cells were incubated in 6-well plate containing 5μM BODIPY-C11 dye (Invitrogen, D3861). After incubation for 20 min, cells were washed with PBS followed by re-suspending in 500 μl of PBS. The cell suspension was filtered through cell strainer (0.4 μm nylon mesh) and subjected to the flow cytometry.
Instrument	BD FACSAria cytometer (BD Biosciences).
Software	BD FACSDiva software to collect data and FlowJo_V7.6 software to analyze data.
Cell population abundance	At least 10000 cells were analyzed for each sample.
Gating strategy	The cells were gated on FSC-H/SSC-H basis on the location known to contain melanoma cells.

- [x] Tick this box to confirm that a figure exemplifying the gating strategy is provided in the Supplementary Information.
